# Data on Mapping 444 Dairy Small Ruminant Farms during a Countrywide Investigation Performed in Greece

**DOI:** 10.3390/ani13122044

**Published:** 2023-06-20

**Authors:** Daphne T. Lianou, Charalambia K. Michael, George C. Fthenakis

**Affiliations:** Veterinary Faculty, University of Thessaly, 43100 Karditsa, Greece

**Keywords:** dairy, goat, mastitis, milk quality, parasitology, sheep

## Abstract

**Simple Summary:**

This study refers to the mapping the dairy sheep and goat industry in Greece, in which information was obtained from 325 sheep and 119 goat farms. The findings can be used as baseline measurements; individual farms and cohorts of farms can be compared against the current results to draw conclusions against the countrywide situation. The results can also be used as reference points for the future, in order to assess changes that might have occurred in the meantime. Finally, the findings can be useful in the health management of small ruminants, in providing evidence-based support, within the scope of health management.

**Abstract:**

The small ruminant industry is the most important branch of Greek agriculture. Nevertheless, despite the importance of small ruminant farming for Greece, no detailed mapping of the respective farms has been undertaken and published. The present work refers to mapping the dairy sheep and goat industry in Greece through an extensive, countrywide cross-sectional study, in which information was obtained from 325 sheep and 119 goat farms. The objectives were the collection, the classification and the presentation of data obtained from all these farms through interviews, using a questionnaire and through examination of samples collected during the visits. All the farms enrolled in the study were visited by the investigators. Initially, information was obtained by means of a detailed, structured questionnaire with 442 questions. Moreover, samples of milk were collected from the bulk-tank of each farm and faecal samples were collected from female animals in each farm. The milk samples were processed for cytological and microbiological examination. *Staphylococcus* spp. and *Listeria* spp. isolates were recovered and identified at a species level; furthermore, a full antibiotic sensitivity pattern assessment was conducted. Faecal samples were processed by standard parasitological tests for the identification of protozoan, trematode, cestode and gastrointestinal and respiratory nematode parasites. The paper presents the cumulative findings of the study, i.e., the answers to the questions during the interviews and the results of the laboratory examinations performed in the samples; the findings are presented separately for sheep and goat farms. The findings can be used as baseline measurements; individual farms and cohorts of farms can be compared against the current results to draw conclusions against the countrywide situation. Moreover, the current results can be used as reference points for the future, in order to assess changes that might have occurred in the meantime. The study also described the differences between farms with sheep or goats; in total, differences in 137 parameters were identified. Some of these can be attributed to the different management systems practiced; sheep flocks are managed mostly under the intensive or semi-intensive system, whilst goat herds are managed mostly under the semi-extensive or extensive system. These findings can be useful in the health management of small ruminants, in providing evidence-based support and within the scope of precise livestock medicine and health management.

## 1. Introduction

Greece has a high number of sheep (8,400,000 animals) and goats (3,600,000 animals) [1]. The relevant populations of these species amount to approximately 6.5% and 22.0% of the respective numbers of small ruminants in Europe [2]. Thus, sheep and goat farming constitutes the most important animal farming industry in Greece. The sector generates 18% of the total gross product of the primary sector and 0.8% of the total annual gross domestic product of the country [3].

Sheep and goats follow a dairy production system in the majority of farms (>98% of such farms in the country) [3]. In 2022, annual milk production from sheep and goats in Greece was 716,000 and 160,000 tons, respectively [4]. It is noteworthy that, in Greece, small ruminant milk production exceeds milk production from cattle [5], which, in 2022, amounted to 643,000 tons [6], and, in this context, Greece is unique in Europe. In Greece (as well as in Europe), the greater proportion (>90%) of the milk collected from small ruminants is used for the manufacturing of dairy products (mainly cheese and yoghurt) [2].

However, and despite the importance of small ruminant farming for Greece, no detailed countrywide mapping of the respective farms has been undertaken and published. Previous papers reported limited information, for example, only regarding specific practices applied in sheep and goat farms or with narrow geographical coverage. For example, previous studies reported information about management practices performed only in farms in some parts of the country (e.g., island of Lesvos: Kizos et al. [7], Peloponnese: Manolopoulou et al. [8], Central Greece: Perucho et al. [9]); other studies reported information only about farms with specific animal breeds (e.g., sheep of the Friesarta breed: Kominakis et al. [10], sheep of the Chios breed: Gelasakis et al. [11]).

Thus far, an extensive investigation of the countrywide coverage in Greece of sheep flocks and goat herds, aiming to assess and evaluate many variables, has not been performed. Such an extensive study can contribute valuably to the small ruminant industry.

In this respect, an extensive cross-sectional study of the dairy sheep and goat industry in Greece was performed. During the study, information was obtained from 325 sheep and 119 goat farms throughout the country. The objectives were the collection, the classification and the presentation of the data obtained from all these farms. Cumulatively, over 215,000 data were collected during the study.

## 2. Materials and Methods

### 2.1. Sheep and Goat Farms and Collection of Information and Samples

**Figure 1:**
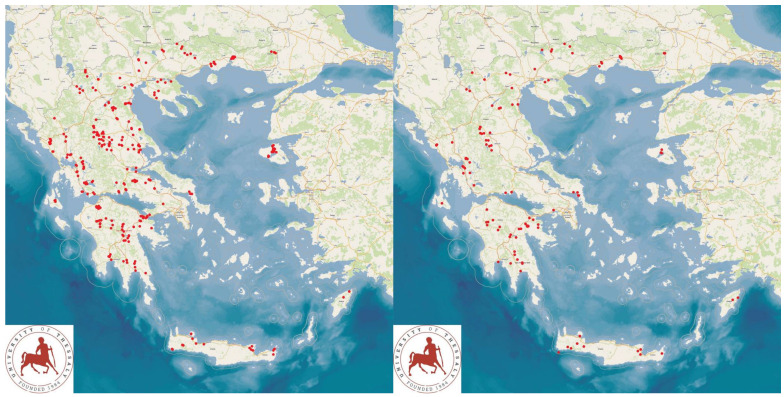
Locations (red dots) of the 325 sheep (**left** figure) and 119 goat (**right** figure) farms around Greece, which were visited for a detailed and extensive interview and for collection of samples.

The study was performed in 325 sheep flocks and 119 goat herds (Figure 1) throughout Greece, during the period April 2019 to July 2020. Initially, professional veterinarians, active in the field of sheep/goat health management practice across Greece, were contacted by telephone and were asked about their interest and willingness to collaborate in the project. Thus, in total, 48 veterinarians were contacted and, among them, 47 (97.9%) agreed to collaborate.

The farms were selected by the collaborating veterinarians and were enrolled in the study on a convenience basis (i.e., the acceptance by farmers to receive a visit by academic veterinary staff for a detailed and extensive interview and for the collection of samples). Each of these veterinarians had a stable, although not contractual, association with the respective farm, among those selected for visitation, and were responsible for their decisions and actions in relation to the health and welfare of the animals therein, in full accordance with the relevant veterinary conduct codes. Farm visits were arranged by the collaborating veterinarians. The three investigators travelled across Greece and personally visited all the farms included in this study, in order to collect information and samples. In total, visits had been scheduled to 446 farms; however, in two farms (0.4%), upon the arrival of the investigators to the respective farms, the farmers declined the visit and the interview and did not agree to collaborate.

Upon arrival at the farm, the veterinarian accompanying the investigators introduced them to the farmer. The senior investigator in the party explained in detail to all the farmers the background, the objectives and the characteristics of the study, as well as the aims of the interview and the sampling procedures; moreover, he introduced the farmer to the two junior investigators.

A structured detailed questionnaire was employed to carry out the interview. This questionnaire had been previously tested for the validity of its content [12]. In the questionnaire, there were general questions, as well as questions about the socio-demographic characteristics of farmers, about the animals, about the health management and the production characteristics of the farm, as well as about the infrastructure [12]. If farmers requested the clarification of the questions asked during the interview, appropriate answers and relevant clarifications were provided immediately by the interviewer. In total, the questionnaire included 442 questions [12].

During the visits to the farms, samples of bulk-tank milk were also collected [13]. For sampling, after stirring the content of the tank, milk samples were collected by means of plastic, sterile pipettes, following the aseptic technique. Four bulk-tank samples were collected from the tank of each farm. Faecal samples were subsequently obtained from the female animals (ewes/does) on each farm [14]. Faecal samples were obtained directly from the rectum of animals, following the standard technique. In each flock or herd, 20, 30, 40, or 50 females were selected for sampling (respectively, for farms with 165, 166–330, 331–500, or >500 ewes/does). Finally, animals in the farm were assessed for body condition; this was performed by a certified European Veterinary Specialist in Small Ruminant Health Management, in order to maintain uniform and consistent scoring results (0–5, including half scores), based on the appropriate published standards [15].

Samples were stored at 0.0 to 4.0 °C (milk) or at 8.0 to 10.0 °C (feces) by using portable refrigerators. Transportation of the samples to the laboratory was made by the investigators and by car; samples collected from farms in the islands were also transported as accompanying luggage by airplane or by boat.

### 2.2. Laboratory Examinations

Milk samples were processed for somatic cell counting and for measuring chemical composition [13], and were performed on the samples within 4 h after collection. From each milk sample obtained, two subsamples were created and processed; therefore, each separate test was performed four times (each one in different subsamples). Somatic cell counting (Lactoscan SCC; Milkotronic Ltd., Nova Zagora, Bulgaria) and measurement of milk composition (Lactoscan Farm Eco; Milkotronic Ltd., Nova Zagora, Bulgaria) were performed on each of the four subsamples [13] within 4 h after sample collection.

Bacteriological examinations started within 24 h after collection of samples. The milk samples were processed for total bacterial count. Total bacterial counts in milk were obtained by following the standardized procedures of the American Public Health Association [16].

Bacteriological examinations were also performed for isolation of *Staphylococcus* spp. (by using standard techniques [17,18]) and for identification of these bacteria on a species level, which was performed by means of Matrix-Assisted Laser Desorption/Ionization Time-of-Flight Mass Spectrometry (VITEK MS; BioMerieux, Marcy-l’-Étoile, France). Examination was also performed for the isolation of *Listeria* spp. (by using the officially acceptable ISO 11290-1:2017 [19], which is the currently valid and standardized protocol for this task) and the identification of the isolated organisms at species level (by using MALDI-TOF as above).

Staphylococcal isolates were tested in vitro for evaluation of potential slime production and biofilm formation. For this, the combination of (a) the appearance of colonies on Congo Red agar plates and (b) the findings of the microplate adhesion test, as detailed by Vasileiou et al. [20], were used.

Staphylococcal isolates were processed for testing antibiotic susceptibility to 20 antibiotics (amikacin, ampicillin, ceftaroline, ciprofloxacin, clindamycin, erythromycin, fosfomycin, fucidic acid, gentamicin, linezolid, moxifloxacin, mupirocin, mupirocin high level, oxacillin, penicillin G, rifampin, teicoplanin, tetracycline, tobramycin and trimethoprim–sulfamethoxazole). Susceptibility testing was carried out by using the automated system BD Phoenix™ M50 (BD Diagnostic Systems, Sparks, MD, USA). The criteria of the European Committee on Antimicrobial Susceptibility Testing (EUCAST) (http://www.eucast.org) were considered for the interpretation of the results.

The susceptibility of the *Listeria* spp. isolates to five antibiotics (benzylpenicillin, ampicillin, meropenem, erythromycin and trimethoprim/sulfamethoxazole) was tested by means of the disk diffusion method, also by following the relevant recommendations of EUCAST.

Parasitological examinations started within 48 h after the collection of samples and were performed as detailed before [13]. In brief, 5 g of each of the individual animal’s faecal sample from a farm were taken initially and mixed to form a pooled faecal sample from the farm, which was then processed in a homogenizing blender. The usefulness of pooling ovine faecal samples, as a rapid procedure for the identification of gastrointestinal helminths at a farm level, has been confirmed by Rinaldi et al. [21].

In the pooled faecal samples, the following parasitological tests were performed: the McMaster technique (3 g), the flotation method (1 g), the sedimentation technique (1 g) and coproculturing (remaining quantity). Also, a faecal smear was performed and stained according to the Ziehl–Neelsen technique for microscopic observation [22]. Each of the first three techniques were applied in quadruplicate samples (each 5 g) obtained from the pooled faecal sample, whilst coproculture was performed once. Parasitological examinations aimed for identification of protozoan, trematode, cestode and gastrointestinal and respiratory nematode parasites.

### 2.3. Data Management and Analysis

Data were curated and processed in Microsoft Excel and analyzed using SPSS v. 21 (IBM Analytics, Armonk, NY, USA). Initially, basic descriptive analyses were carried out and descriptive statistical measures appropriate for each type of data were obtained. Results obtained from sheep flocks and goat herds were considered separately for the presentation of the findings and the analysis. Moreover, in order to evaluate potential associations with the location of farms, the 13 administrative regions of Greece were clustered into four main areas: North, Central, South and Islands (Table S1, Figure S1).

Comparisons between the results obtained for sheep flocks and goat herds were performed using Pearson’s chi-squared test, Fisher exact test, z-test for proportions, analysis of variance or Kruskal–Wallis test, as appropriate. For results of somatic cell counts and total bacterial counts in milk, appropriate logarithmic transformations were performed before the analysis [23]. In all analyses, statistical significance was defined at *p* < 0.05.

## 3. Results

**Figure 2:**
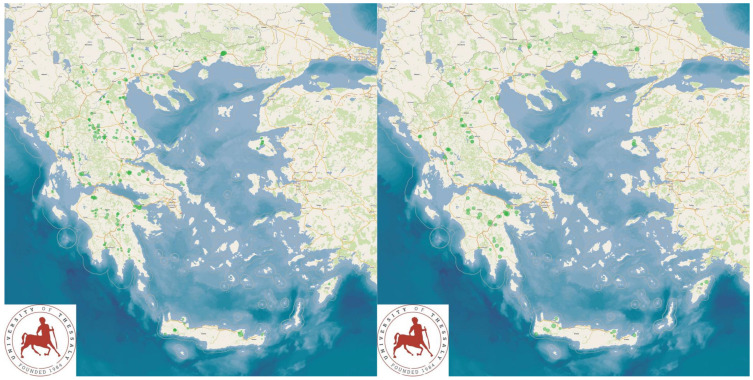
Locations (green dots) of 325 sheep flocks (**left** figure) and 119 goat herds (**right** figure) around Greece, in accordance with somatic cell counts in bulk-tank milk (diameter of dots on maps is proportionate to cell counts).

**Figure 3:**
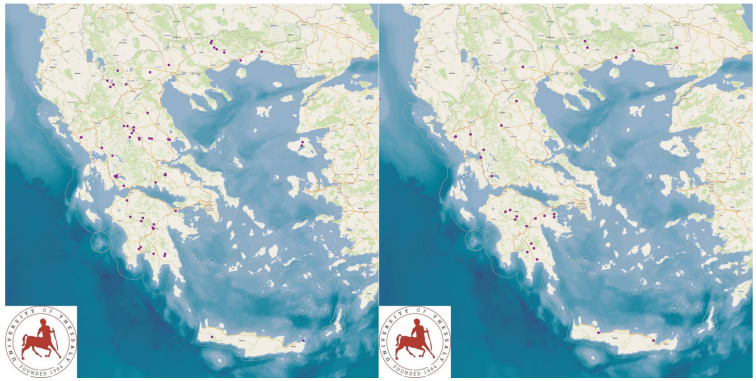
Locations (purple dots) of sheep flocks (**left** figure) and goat herds (**right** figure) around Greece, in which total bacterial counts over 1500 × 10^3^ cfu mL^−1^ in bulk-tank milk were detected.

**Figure 4:**
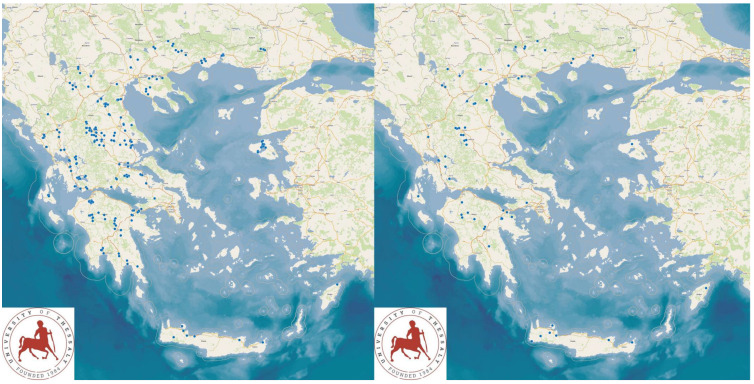
Locations (blue dots) of sheep flocks (**left** figure) and goat herds (**right** figure) around Greece, in which machine-milking was practiced.

**Figure 5:**
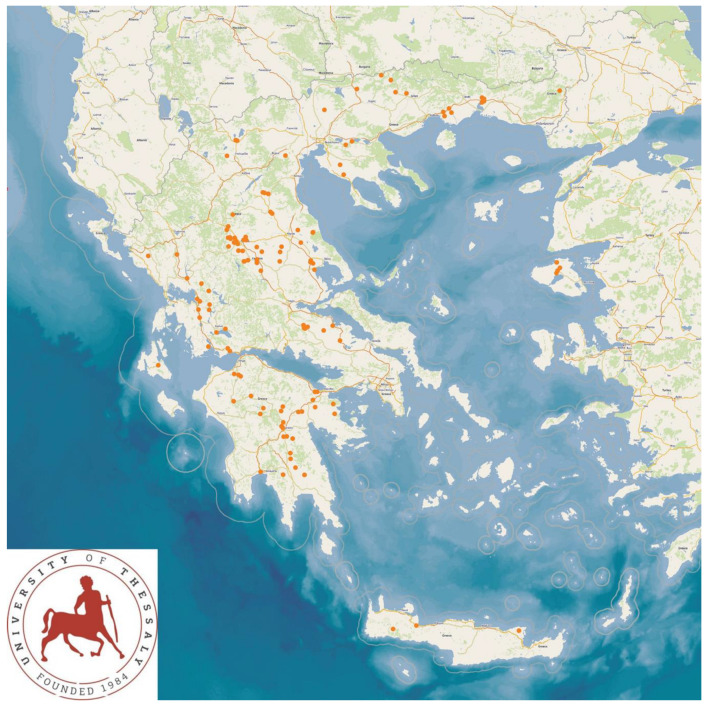
Locations (orange dots) of sheep flocks and goat herds around Greece, in which staphylococci resistant to at least one antibiotic were isolated from bulk-tank milk.

**Figure 6:**
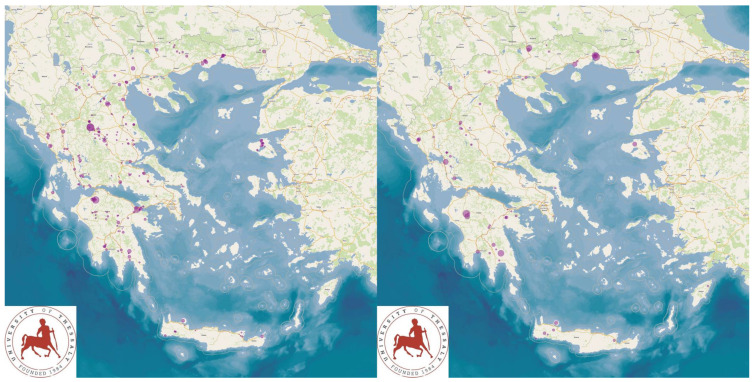
Locations (purple dots) of sheep flocks (**left** figure) and goat herds (**right** figure) around Greece, in accordance with the incidence of clinical mastitis in ewes/does (diameter of dots on maps is proportionate to incidence of the pathological condition).

**Figure 7:**
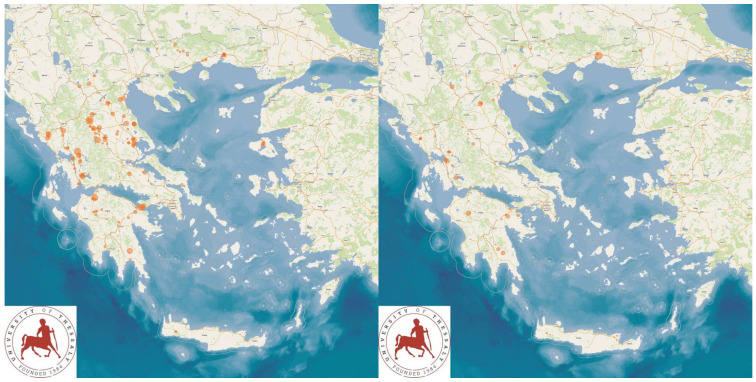
Locations (orange dots) of sheep flocks (**left** figure) and goat herds (**right** figure) around Greece, in accordance with incidence of diarrhoea in lambs/kids (diameter of dots on maps is proportionate to incidence of the pathological condition.

**Figure 8:**
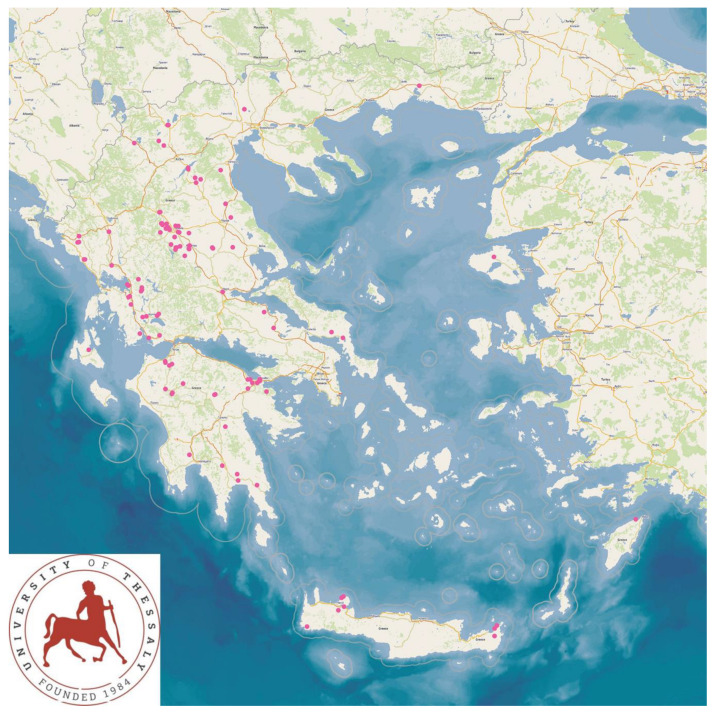
Locations (pink dots) of sheep flocks and goat herds around Greece, in which reproductive control techniques were employed.

**Figure 9:**
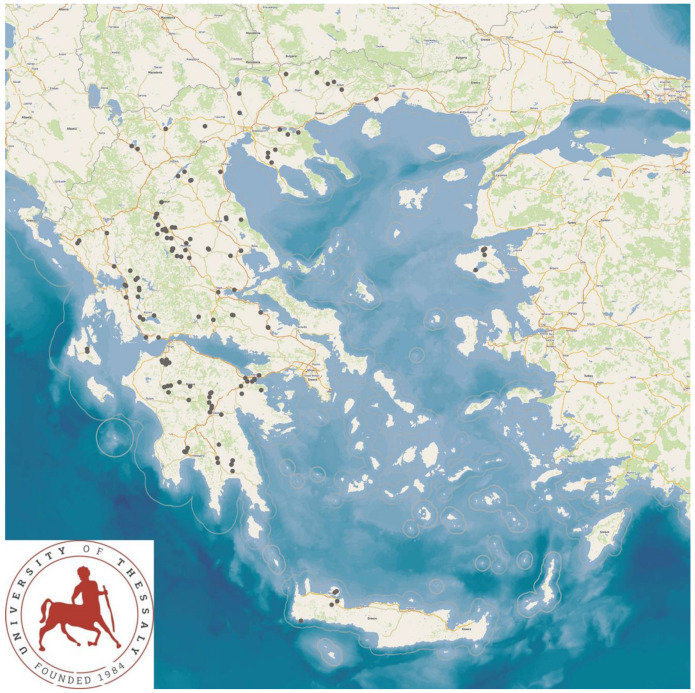
Locations (black dots) of sheep flocks and goat herds around Greece, in which vaccination against staphylococcal mastitis was performed.

**Figure 10:**
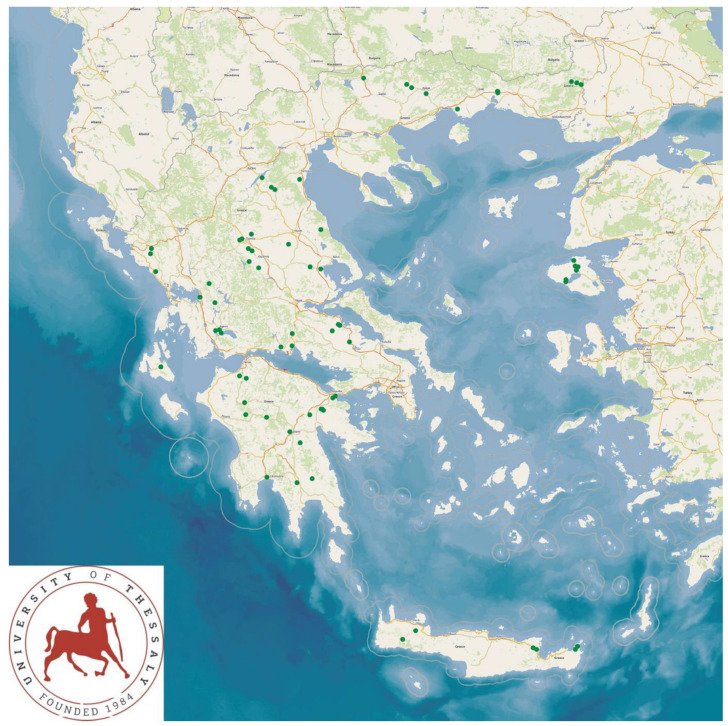
Locations (green dots) of sheep flocks and goat herds around Greece, in which *D. dendriticum* was detected in pooled faecal samples.

**Figure 11:**
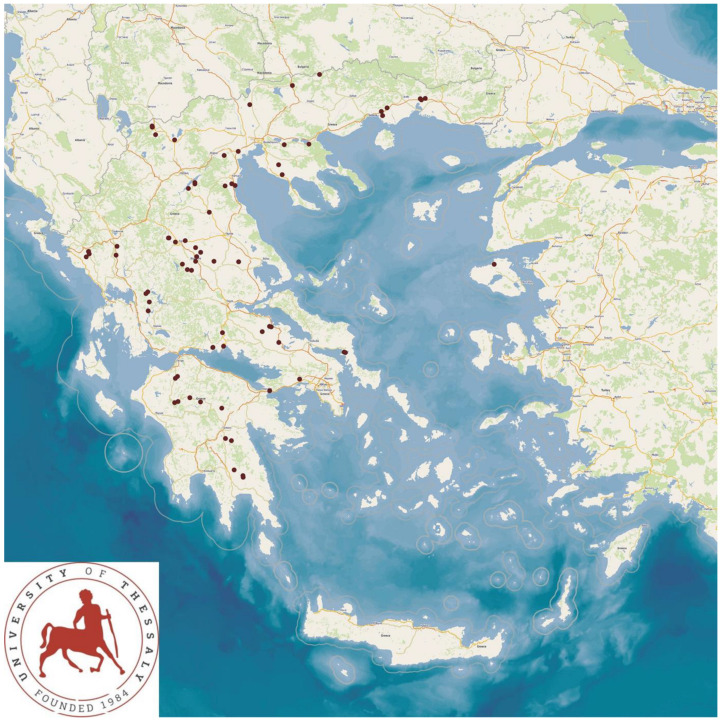
Locations (brown dots) of sheep flocks and goat herds around Greece, in which *Nematodirus* spp. was detected in pooled faecal samples.

**Figure 12:**
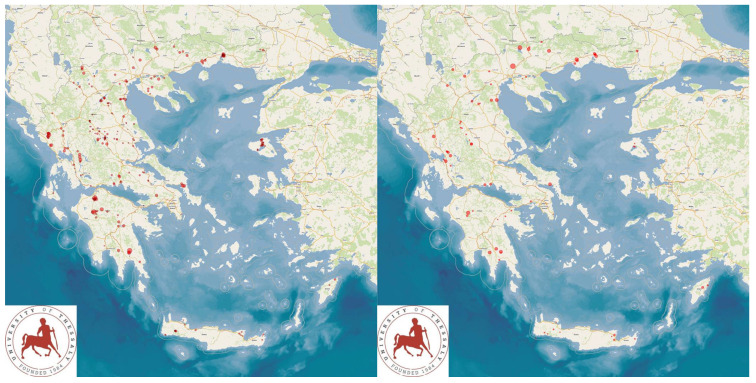
Locations (red dots) of 280 sheep flocks (**left** figure) and 90 goat herds (**right** figure) around Greece, in which no anthelmintic treatment was carried out during the two months prior to sampling, in accord with epg counts in pooled faecal samples (diameter of dots on maps is proportionate to epg counts).

The study presents the detailed results for small ruminant dairy farms, sheep flocks (*n* = 325) and goat herds (*n* = 119), as obtained from farms located in all the 13 administrative regions of Greece. The farms studied in the present work represented 0.84% and 0.91% of the total sheep flocks and goat herds, respectively, which delivered milk at the time of the investigation [4]. The farms from which the data were obtained included, in total, 110,228 sheep and 30,192 goats. In total, over 215,000 data were collected. It is noted that, for their collection, approximately 35,000 km in total were driven across Greece and, additionally, six domestic flights and six domestic sails were also taken. The cumulative findings are in Tables S2 and S3. Selected variables among those findings are shown in Table 1 and Table 2 and in Figure 1, Figure 2, Figure 3, Figure 4, Figure 5, Figure 6, Figure 7, Figure 8, Figure 9, Figure 10, Figure 11 and Figure 12.

There was a seasonality in the visits to the farm (Table S4), with a significantly smaller proportion of farms visited during the autumn (*p* < 0.0001), although there was no difference in the proportion of farms visited during each of the other three seasons (*p* = 0.07).

**Figure 13:**
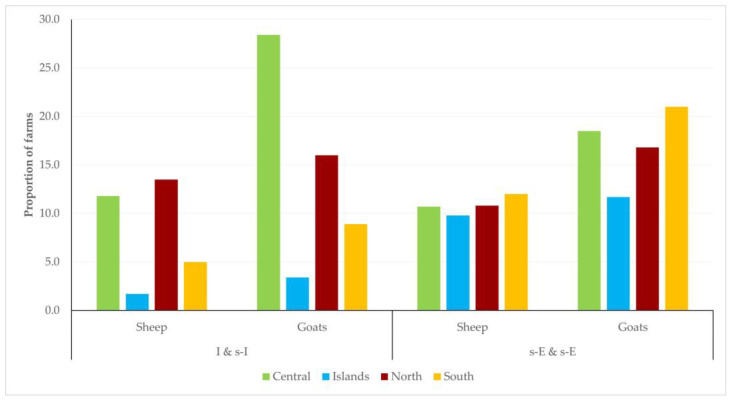
Management system applied in 444 small ruminant farms in a countrywide investigation in Greece, in accordance with the location of the farms.

There was clear evidence of significant differences in the distribution of farms in the country in accordance with the management system and the geographical area (*p* < 0.0001 for sheep flocks and *p* = 0.010 for goat herds). Most farms managed under the intensive or semi-intensive system were located in the central or north areas of the country, whilst farms managed under the semi-extensive or extensive system were, in general, equally distributed among the four geographical areas (Figure 13, Table S5).

**Figure 14:**
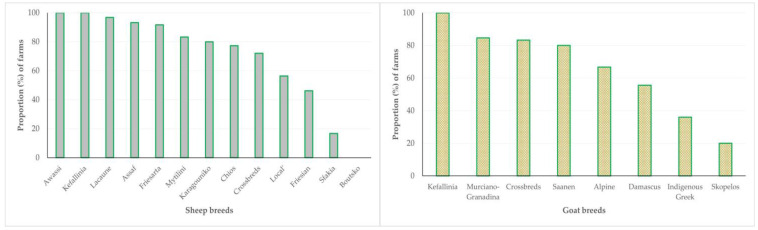
Proportion of 444 small ruminant farms applying machine-milking in accordance with the breed of animals in the farms, as found in a countrywide investigation in Greece.

There were significant associations between the management system applied in the farms and the breed of animals therein (*p* < 0.0001) (Table S6). The results indicated that breeds characterized by high milk production (e.g., sheep: Chios, Lacaune; goats: Murciano-Granadina, Saanen) were more frequently present in farms managed under the intensive or semi-intensive system, whilst breeds characterized by low milk production were more frequently present in farms managed under the semi-extensive or extensive system. Furthermore, in farms with breeds with milk production, machine-milking was applied more frequently than in farms with low milk production (*p* < 0.001 for sheep flocks, *p* = 0.002 for goat herds) (Figure 14, Table S7).

The study also described differences between farms with sheep or goats. In total, differences in 137 parameters were identified between sheep flocks and goat herds (Appendix A and Appendix B).

Finally, there was a clear association between the breeds of animals in sheep flocks and the geographical location of the farms (*p* < 0.0001) (Table S8). Breeds with high milk production were located mainly in the central and north part of the country. In contrast, no such association was seen for goat herds (*p* = 0.07).

## 4. Discussion

The study presents, for the first time in Greece, the detailed results of dairy small ruminant farms with sheep flocks and goat herds. Moreover, to the best of our knowledge, also at international level, this study is one of the largest studies in small ruminant farms ever performed in any country.

In this study, dairy farms in all parts of Greece, and in all the 13 administrative regions of the country, were visited and included herein. Therefore, the conditions applied throughout the country were considered and assessed and, thus, local and regional conditions, factors and particularities weighed less. That way, there was also some stratification in the selection of the farms in the study, as the flocks and herds visited were located in all the 13 administrative regions of the country.

Although farms were enrolled in the study on a convenience basis, this approach nevertheless guaranteed visit acceptance by the farmers and a lack of suspiciousness and distrust for the investigators, resulting in a relaxed interview. Furthermore, our approach allowed the inclusion of flocks and herds with farmers genuinely willing to participate in the study and to provide thoughtful and correct answers. Moreover, and in order to minimize possible bias, the study also used consistent methodologies.

The visits to the farms had to take place during the milking period of the ewes and does, in order to collect samples of bulk-tank milk for laboratory examinations (Table S2). Lambing/kidding of small ruminants in Greece takes place during the autumn (on average, the mating season in Greece starts in May or June [25]), hence only a smaller proportion of farms could have been visited during that season; this was reflected in the significantly smaller proportion of farms visited in autumn. Nevertheless, there was no significant difference in the proportions of farms visited during the other three seasons, which further explains the reason for the reduced proportions of visits to farms during the autumn.

Previous papers discussed associations and interactions between the various variables and focused on establishing predictors for various outcomes related to these results, e.g., [25,26,27,28,29]. The present paper presents the entirety of the findings, thus showing details of the sheep and goat industries in Greece and allowing overall comparisons with the respective animal industries of other countries and regions of the world. Moreover, the current results can be used by farmers, veterinarians, technical advisors, authorities, etc., as baseline measurements, as they cover an important proportion of the respective sector of the country. Furthermore, the findings and outcomes in individual farms or cohorts of farms (e.g., farms in agricultural cooperatives) can be compared against the current findings; that way, conclusions can be drawn regarding the standing of these farms against the national situation.

Moreover, the current results can be used as reference points for the future. Previously, we considered older studies on the milk quality of sheep, performed in the 1990s in the country, for comparisons against the current findings and assess the potential changes; the average values for somatic cell counts in the bulk-tank milk from sheep flocks in the 1990s were reported to exceed 1.0 × 10^6^ cells mL^−1^ [30,31], whilst the mean value in the current study was found to be 0.488 × 10^6^ (95% confidence intervals: 0.451 × 10^6^–0.529 × 10^6^) cells mL^−1^. Although those studies included a small number of farms and were of limited geographical coverage, they have made possible a comparison of the present situation with that which was prevalent in the country some years ago, even if it is difficult to make direct comparisons between studies that were performed using different methodologies and over a lengthy span of years. The comparison indicated a clear improvement during the last 25 to 30 years. In the same context, the current findings can be used by future researchers, who will study the situation in the country in 15 to 20 years’ time and will compare it to previous relevant studies to assess the changes that will have taken place during those years.

Some of the differences identified between sheep flocks and goat herds can be attributed to the different management systems practiced between the two types of farms: sheep flocks are managed mostly under the intensive or semi-intensive systems, whilst goat herds are managed mostly under the semi-extensive or extensive systems. Inputs in goat farming are lower than in sheep farming, and this is reflected in the infrastructure (e.g., less frequent use of machine-milking, less frequent connection to the national electricity network and larger areas for grazing) and the animals (e.g., smaller size of farms, lower replacement rate) in the farms. The above are reflected in the production characteristics (e.g., smaller number of newborns produced in goat herds, with lower bodyweight at slaughter) and health problems (e.g., higher incidence rate of deaths, higher rate of attacks by wildlife), although there are cases where the extensive type of management seems to be beneficial for the animals (e.g., lower annual incidence of clinical mastitis).

The associations observed between the management system and the geographical location of the farms reflect the general structure of the country. As a general model, farms following the intensive or semi-intensive management system are primarily based in the mainland of the country, near locations that produce the raw material for feedstuff, e.g., cereals and hay (which are cultivated generally in the large plains of the country in Thessaly, central Greece, or Thrace, northern Greece), or near locations of the large feedstuff factories (e.g., central Macedonia, Thessaly). Such farms are high-input agricultural enterprises (e.g., with extensive infrastructure, employment of additional staff) [29] and thus include animals of high-production breeds, in order to achieve profitability. In contrast, farms following the semi-extensive or extensive management system are located in the uplands of the mainland of the country or in the islands, where the availability of feedstuffs would be more costly (due to transportation expenses) or prime land would be expensive to occupy (due to higher margins if made available for other uses, e.g., for the tourist sector). These farms are generally low-input enterprises, in which low milk production by animals on the farm can still contribute to making the enterprise cost-effective, due to the reduced costs and the payment of subsidies.

## 5. Conclusions

The study presents, for the first time, detailed and extensive results for dairy sheep and goat farms. The investigation was based on interviews carried out with respective farmers, using a detailed questionnaire to gather data regarding the situation in the farms, and on the examination of samples collected during the farm visits. In all, the findings can be useful in the health management of small ruminants and in providing evidence-based support within the scope of precise livestock medicine and health management. The data contribute to the understanding of sheep/goat farming systems in Greece, to providing information about the situation in the farms, to providing efficiency, to supporting the development of relevant technologies and to optimizing management practices. Further work from our group, along with the work of other researchers in Greece and internationally, may ultimately thus create a vision for the future of the small ruminant industry.

## Figures and Tables

**Table 1:** Selected cumulative data collected during the mapping of 444 small ruminant farms in a countrywide investigation in Greece, classified according to animal species in the farms.

	Sheep Flocks (*n* = 325)	Goat Herds (*n* = 199)	*p*-Value ^1^
General Details			
Management system ^2^	Intensive: 13.5%, semi-intensive: 43.1%, semi-extensive: 35.7%, extensive: 7.7%	Intensive: 7.5%, semi-intensive: 24.4%, semi-extensive: 51.3%, extensive: 16.8%	<0.0001 ^3^
Infrastructure			
Year of the initial establishment of the farm	*X*: year 1981 ± 1 year ^3^	*X*: year 1977 ± 1 year	0.09
Availability of a main building for animals	Yes: 97.8%, no: 2.2% ^4^	Yes: 98.3%, no: 1.7%	0.75
Availability of a milking parlour	Yes: 78.5%, no: 21.5%	Yes: 55.5%, no: 44.5%	<0.0001
Type of milking parlour	Fishbone: 0.4%, circular: 3.9%, linear parallel: 35.7%, linear one-sided: 59.6%, other: 0.4%	Fishbone: 0.0%, circular: 6.1%, linear parallel: 31.8%, linear one-sided: 62.1%, other: 0.0%	0.85
Number of animal positions in the parlour	*X*: 25 ± 1 positions	*X*: 25 ± 2 positions	0.65
Total number of feed troughs available	*X*: 28 ± 2 troughs	*X*: 26 ± 3 troughs	0.71
Total number of drinking points available	*X*: 10 ± 0.5 points	*X*: 9 ± 1.0 points	0.46
Total grazing land	*X*: 510 ± 50 acres	*X*: 1322 ± 390 acres	0.002
Animals			
No. of female animals (small ruminants) in the farm	*X*: 325 ± 13 ewes	*X*: 237 ± 20 does	0.0006
No. of male animals (small ruminants) in the farm	*X*: 15 ± 0.5 rams	*X*: 16 ± 1.5 bucks	0.47
Breed of female animals	Assaf: 9.2%, Awassi: 0.3%, Boutsko: 0.6%, Chios: 13.5%, Crossbreds: 13.2%, Friesarta: 3.7%, Friesian: 4.0%, Karagouniko: 1.5%, Kefallinia: 0.3%, Lacaune: 29.2%, ‘Local’: 16.9%, Mytilini: 5.5%, Sfakia: 1.8%	Alpine: 7.6%, Crossbreds: 15.1%, Damascus: 15.1%, Kefallinia: 0.8%, Indigenous Greek (*Capra prisca*): 42.0%, Murciano-Granadina: 10.9%, Saanen: 4.2%, Skopelos: 4.2%	n/a
Average age of culling ewes/does	*X*: 5.9 ± 0.1 years	*X*: 6.9 ± 0.1 years	<0.0001
Production Characteristics			
Month of the start of the lambing/kidding season	October (January–December) ^5^	October (January–December)	0.11
Annual milk quantity per animal obtained during the preceding milking period	*X*: 207 ± 5 L	*X*: 201 ± 10 L	0.55
Total number of lambs/kids born per female animal during the preceding lambing/kidding season	*X*: 1.33 ± 0.1 lambs	*X*: 1.30 ± 0.2 kids	0.15
Average age of lambs/kids at slaughter	*X*: 50 ± 1 days	*X*: 65 ± 3 days	<0.0001
Average carcass weight of lambs/kids at slaughter	*X*: 10.0 ± 0.1 kg	*X*: 9.4 ± 0.2 days	0.012
Health Management			
The two health problems in lambs/kids considered to be of higher importance (top three outcomes presented)	Diarrhoea: 71.7%, pneumonia: 27.4%, contagious ecthyma: 7.7%	Diarrhoea: 69.7%, pneumonia: 22.7%, clostridial infection: 10.1%	n/a
The two health problems in adult animals considered to be of higher importance (top three outcomes presented)	Mastitis: 66.2%, pneumonia: 17.5%, lameness: 6.2%	Mastitis: 42.9%, paratuberculosis: 19.3%, pneumonia: 14.3%	n/a
Incidence rate of total deaths, of any cause, in adult animals during the preceding season	5.2% (5.0–5.5%) ^6^	5.9% (5.8–6.0%)	0.023
Collaboration with a veterinarian	Yes: 87.1%, no: 12.9%	Yes: 84.9%, no: 15.1%	0.55
Use of laboratory diagnostic examinations	Yes: 40.9%, no: 59.1%	Yes: 43.7%, no: 56.3%	0.60
Maintenance of prescribed withdrawal periods after administration of pharmaceuticals	Yes: 98.8%, no: 1.2%	Yes: 98.3%, no: 1.7%	0.72
Overall incidence of mastitis during the preceding season	3.9% (3.8–4.0%)	2.8% (2.6–3.0%)	<0.0001
Overall incidence of abortion during the preceding season	2.0% (1.9–2.1%)	2.5% (2.7–2.9%)	<0.0001
Overall incidence of lameness during the preceding season	2.8% (2.7–2.9%)	1.2% (1.1–1.4%)	<0.0001
Overall incidence of respiratory problems in young animals during the preceding season	1.4% (1.3–1.5%)	1.1% (1.0–1.2%)	<0.0001
Overall incidence of diarrhoea in young animals during the preceding season	7.9% (7.8–8.0%)	10.4% (10.0–10.7%)	<0.0001
Reproductive management	No hormonal control: 66.8%, administration of melatonin: 7.1%, administration of progestogens: 27.4%	No hormonal control: 83.2%, administration of melatonin: 4.2%, administration of progestogens: 13.4%	0.0007
Duration of mating period	2 (1–12) months	2 (1–12) months	0.09
Age for lamb/kid removal from their dams	*X*: 50 ± 1 days	*X*: 65 ± 3 days	<0.0001
Daily number of milking sessions	2 (1–3)	2 (1–3)	0.0001
Duration of the dry-period	*X*: 3.0 ± 0.1 months	*X*: 2.9 ± 0.1 months	0.84
Vaccination against *Chlamydia* infection	Yes: 40.0%, no: 60.0%	Yes: 32.8%, no: 67.2%	0.16
Vaccination against *Brucella* infection	Yes: 100.0%, no: 0.0%	Yes: 100.0%, no: 0.0%	n/a
Vaccination against clostridial infection	Yes: 97.2%, no: 2.8%	Yes: 99.2%, no: 0.8%	0.23
Vaccination against mastitis	Yes: 39.7%, no: 61.2%	Yes: 28.6%, no: 71.4%	0.047
Vaccination against contagious agalactia	Yes: 57.2%, no: 42.8%	Yes: 54.6%, no: 45.4%	0.62
Vaccination against bacterial respiratory infections	Yes: 44.3%, no: 55.7%	Yes: 32.8%, no: 67.2%	0.028
Vaccination against paratuberculosis	Yes: 3.4%, no: 96.6%	Use: 26.1%, no: 73.9%	<0.0001
Administration of anthelmintics to sheep/goats in the farm	Yes: 99.1%, no: 0.9%	Yes: 98.3%, no: 1.7%	0.50
Administration of ectoparasiticides to sheep/goats in the farm	Yes: 33.5%, no: 66.5%	Yes: 58.0%, no: 42.0%	<0.0001
Application of disinfections in the farm	Yes: 91.1%, no: 8.9%	Yes: 85.7%, no: 14.3%	0.10
Foot care	Yes: 68.9%, no: 31.1%	Yes: 60.5%, no: 39.5%	0.10
Nutrition			
Provision of hay as fodder to animals	Yes: 99.7%, no: 0.3%	Yes: 97.5%, no: 2.5%	0.029
Total quantity of hay consumed during the preceding season	*X*: 84 ± 5 tons	*X*: 46 ± 7 tons	0.0003
Provision of straw to animals	Yes: 79.4%, no: 20.6%	Yes: 65.5%, no: 34.5%	0.003
Provision of silage to adult animals	Yes: 22.2%, no: 77.8%	Yes: 15.1%, no: 84.9%	0.10
Total quantity of silage consumed during the preceding season	*X*: 142 ± 31 tons	*X*: 98 ± 35 tons	0.50
Provision of finished feed to animals	Yes: 93.5%, no: 6.5%	Yes: 86.6%, no: 13.4%	0.018
Total quantity of finished feed (concentrate) consumed during the preceding season	*X*: 86 ± 6 tons	*X*: 66 ± 8 tons	0.12
Premix purchase for use in diets	Yes: 88.0%, no: 12.0%	Yes: 81.5%, no: 18.5%	0.08
Human Resources			
Age	*X*: 47 ± 1 years	*X*: 47 ± 1 years	0.64
Length of previous animal farming experience	*X*: 24 ± 1 years	*X*: 25 ± 1 years	0.80
Highest level of general education	Primary: 17.5%, secondary: 69.2%, tertiary: 13.2%	Primary: 16.8%, secondary: 74.8%, tertiary: 8.4%	0.35
Farmer by profession	Yes: 89.8%, no: 10.2%	Yes: 88.2%, no: 11.8%	0.63
Personal opinion regarding occurrence of transmission of diseases from animals to the farmer or members of the family	Yes: 13.5%, no: 86.5%	Yes: 22.7%, no: 77.3%	0.020
Farm worker employment	Yes: 37.8%, no: 62.2%	Yes: 28.6%, no: 71.4%	0.07

^1^ *p*-value for comparison between sheep flocks and goat herds; ^2^ Management system classified according to the system of the European Food Safety Authority [24]; ^3^ Figures present mean ± standard error of the mean (*X* ± σ_M_.); ^4^ Figures present proportions of farms for each category within the variable; ^5^ Figures present median (minimum–maximum) value; ^6^ Figures present average (95% confidence interval) value.

**Table 2:** Selected cumulative results obtained during field and laboratory examinations in samples collected from 444 small ruminant farms in a countrywide investigation in Greece, classified according to animal species in the farms.

	Sheep Flocks (*n* = 325)	Goat Herds (*n* = 199)	*p*-Value ^1^
Clinical Examinations of Animals at the Farms			
Body condition scoring	*X*: 2.38 ± 0.02 (scale: 0–5) ^2^	*X*: 2.54 ± 0.03 (scale: 0–5)	<0.0001
Laboratory Examinations in Bulk-Tank Milk Samples			
Somatic cell counts	0.488 × 10^6^ (0.451 × 10^6^–0.529 × 10^6^) cells mL^−1 3^	0.838 × 10^6^ (0.759 × 10^6^–0.933 × 10^6^) cells mL^−1^	<0.0001
Total bacterial counts	398 × 10^3^ (331 × 10^3^–479 × 10^3^) cfu ^4^ mL^−1^	581 × 10^3^ (447 × 10^3^–741 × 10^3^) cfu mL^−1^	<0.0001
Staphylococcal isolation	Yes: 63.4%, no: 36.6% ^5^	Yes: 63.0%, no: 37.0%	0.94
Listeria isolation	Yes: 1.2%, no: 98.8%	Yes: 0.0%, no: 100.0%	0.22
Fat content	*X*: 6.16% ± 0.05%	*X*: 4.77% ± 0.44%	0.0005
Protein content	*X*: 4.43% ± 0.01%	*X*: 3.23% ± 0.30%	0.0008
Parasitological Examinations in Faecal Samples			
epg counts	*X*: 214 ± 13	*X*: 219 ± 22	0.77

^1^ *p*-value for comparison between sheep flocks and goat herds; ^2^ Figures present mean ± standard error of the mean (*X* ± σ_M_.); ^3^ Figures present mean (95% confidence intervals); ^4^ cfu: colony-forming units; ^5^ Figures present proportions of farms for each category within the variable.

## Data Availability

All data presented in this study are in the Supplementary Materials.

## Supplementary Materials

The following supporting information can be downloaded at: https://www.mdpi.com/article/10.3390/ani13122044/s1, Table S1: Geographical areas of Greece (*n* = 4), in which administrative regions and regional units of the country were clustered for characterizing the locations of 444 small ruminant farms in a countrywide investigation in Greece; Figure S1: Map of Greece indicating the geographic areas of the country (*n* = 4), in which administrative regions and regional units of the country were clustered for characterizing the locations of 444 small ruminant farms in a countrywide investigation in Greece; Table S2: Cumulative findings of all data collected during the mapping of 444 small ruminant farms in a countrywide investigation in Greece, classified according to animal species in the farms; Table S3: Cumulative findings of all results obtained during field and laboratory examinations in samples collected from 444 small ruminant farms in a countrywide investigation in Greece, classified according to animal species in the farms; Table S4: Season of the year during which visits were made to 444 small ruminant farms in a countrywide investigation in Greece; Table S5: Management system applied in 444 small ruminant farms in a countrywide investigation in Greece in accordance with the location of the farms; Table S6: Breed of ewes and does in 444 small ruminant farms in a countrywide investigation in Greece in accordance with the management applied therein; Table S7: Breed of ewes and does in 444 small ruminant farms in a countrywide investigation in Greece in accordance with the milking mode applied in the farms; Table S8: Breed of ewes and does in 444 small ruminant farms in a countrywide investigation in Greece in accordance with the location of the farms.

## Appendix A

**Table A1:** Differences observed in parameters (*n* = 130) between sheep flocks and goat herds in the cumulative results of data obtained during the mapping of 444 small ruminant farms in a countrywide investigation in Greece.

	Sheep Flocks (*n* = 325)	Goat Herds (*n* = 199)	*p*-Value ^1^
General Details			
Management system ^2^	Intensive: 44, semi-intensive: 140, semi-extensive: 116, extensive: 25 ^3^	Intensive: 9, semi-intensive: 29, semi-extensive: 61, extensive: 20	<0.0001
Infrastructure			
General information			
Accessory building(s) for animals	Yes: 222, no: 103	Yes: 93, no: 26	0.043
Availability of a milking parlour	Yes: 255, no: 70	Yes: 66, no: 53	<0.0001
Availability of a waiting area before the milking parlour	Yes: 226, no: 29	Yes: 64, no: 2	0.041
Availability of personnel areas	Yes: 179, no: 145	Yes: 40, no: 79	0.009
Availability of an office	Yes: 128, no: 197	Yes: 34, no: 85	0.036
Availability of a lavatory	Yes: 143, no: 182	Yes: 38, no: 81	0.022
Availability of footbath	Yes: 45, no: 280	Yes: 10, no: 109	0.005
Availability of isolation facilities for animals	Yes: 247, no: 78	Yes: 77, no: 42	0.018
Availability of access road to the farm	Yes: 251, no: 74	Yes: 78, no: 41	0.013
Electricity source	National network: 253, diesel generator: 48, own solar cells: 0	National network: 72, diesel generator: 29, own solar cells: 3	0.0002
Main building			
Material of the walls	Cinder blocks: 130, tin: 99, bricks: 69, panels: 44, wood: 21, cement: 7, stone: 5, canvas: 4, nylon: 4, plaster: 1, plastic: 1	Cinder blocks: 57, tin: 38, bricks: 19, panels: 13, wood: 8, cement: 2, stone: 7, canvas: 0, nylon: 1, plaster: 0, plastic: 0	stone: 0.014 all others: 0.08
Opening in the roof	Yes: 234, no: 84	Yes: 35, no: 82	<0.0001
Material of the floor	Soil: 293, concrete: 29, stone: 16, slatted metal: 2, slatted wood: 2, slatted plastic: 1, wood: 1	Soil: 105, concrete: 12, stone: 1, slatted metal: 1, slatted wood: 2, slatted plastic: 0, wood: 2,	stone: 0.0002, wood: 0.015, all others: >0.22
Availability of straw bedding	Yes: 268, no: 60	Yes: 76, no: 41	0.0002
Annual frequency of removal/clean-up of the straw bedding	*X*: 3 ± 0.5 occasions ^4^	*X*: 5 ± 0.5 occasions	0.003
Availability of ventilators	Yes: 47, no: 281	Yes: 8, no: 109	0.035
Availability of artificial lighting	Yes: 278, no: 40	Yes: 83, no: 34	<0.0001
Building for lambs/kids			
Availability of milk replacer facilities	Yes: 22, no: 303	Yes: 1, no: 118	0.013
Openings in the walls	Yes: 28, no: 215	Yes: 1, no: 85	0.004
Opening in the roof	Yes: 28, no: 215	Yes: 1, no: 85	0.004
Milking parlour			
Year of initial establishment	*X*: year 2010 ± 0.5 year	*X*: year 2012 ± 0.5 year	0.030
System check-ups performed by technicians	Yes: 219, no: 36	Yes: 50, no: 16	0.047
Availability of a milk tank	Yes: 307, no: 18	Yes: 103, no: 16	0.006
Equipment			
Scale type available	Large: 114, portable: 140	Large: 28, portable: 59	<0.015
Availability of a roller crusher	Yes: 163, no: 162	Yes: 40, no: 79	0.002
Availability of a feed mill	Yes: 134, no: 191	Yes: 34, no: 85	0.015
Availability of automatic water filling system in troughs	Yes: 167, no: 158	Yes: 40, no: 79	0.0009
Availability of a tractor	Yes: 235, no: 90	Yes: 70, no: 49	0.007
Land			
Grazing practiced	Yes: 281, no: 44	Yes: 112, no: 7	0.025
Total grazing land	*X*: 510 ± 50 acres	*X*: 1322 ± 390 acres	0.002
Ownership of the grazing land	Farmer’s: 225, other private: 175, public: 103	Farmer’s: 86, other private: 55, public: 58	public: 0.003, other private: 0.01, farmer’s: >0.20
Private grazing land	*X*: 152 ± 18 acres	*X*: 343 ± 113 acres	0.018
Irrigation of the private grazing land	Yes: 123, no: 115	Yes: 27, no: 65	0.0003
Plant types available in the grazing land	Oat: 151, clover: 55, ryegrass: 53, barley: 50, vetch: 31, corn: 13, sorghum: 10, alfalfa: 6, rye: 4, olive trees: 3, spurges: 2, poppies: 1, wild grass: 1, wheat: 0	Oat: 44, clover: 15, ryegrass: 16, barley: 28, vetch: 8, corn: 8, sorghum: 3, alfalfa: 2, rye: 3, olive trees: 0, spurges: 0, poppies: 0, wild grass: 1, wheat: 7	oat: 0.005, all others: >0.05
Animals			
Small ruminants			
No. of female animals	*X*: 325 ± 13 ewes	*X*: 237 ± 20 does	0.0006
Average age of culling ewes/does	*X*: 5.9 ± 0.1 years	*X*: 6.9 ± 0.1 years	<0.0001
Average age of culling rams/bucks	*X*: 4.4 ± 0.2 years	*X*: 4.9 ± 0.2 years	0.009
Average annual replacement rate of ewes/does	*X*: 17.0% ± 0.2%	*X*: 14.5% ± 0.3%	<0.0001
Average annual replacement rate of rams/bucks	*X*: 22.5% ± 1.0%	*X*: 20.5% ± 1.0%	0.009
Source of replacement animals	Own animals: 165, purchase: 24, both sources: 136	Own animals: 77, purchase: 6, both sources: 35	0.026
Other domestic animals in the farm			
Dogs	*X*: 5.0 ± 0.5 animals	*X*: 7.0 ± 0.5 animals	0.001
Sheepdogs	*X*: 4.0 ± 0.5 animals	*X*: 6.0 ± 0.5 animals	0.0001
Avian wildlife			
Avian wildlife identified	*Accipiter brevipes*: 10, *Anas platyrhynchos*: 18, *Anser* spp.: 2, *Aquila chrysaetos* ^ *Aquila nipalensis* ^ *Aquila* *fasciata*: 67, Ardeidae family: 3, *Athene noctua*: 5, *Bubo bubo*: 5, *Carduelis carduelis*: 2, *Ciconia ciconia*: 9, *Columba livia*: 146, *Columba palumbus*: 4, *Corvus corax*: 79, *Corvus cornix*: 10, *Coturnix coturnix*: 8, *Cuculus canorus*: 1, *Cygnus cygnus*: 2, *Erithacus rubecula*: 1, *Falco* spp.: 149, *Fringillia coelebs*: 3, *Galerida crisata*: 2, *Garrulus glanda-rius*: 26, *Gypaetus barbatus*: 3, *Gyps* spp.: 12, *Hirundo rustica*: 179, Laridae family: 11, *Luscinia megarhynchos*: 1, Passeridae family: 143, *Pelecanus onocrotalus*: 2, *Perdix perdix*: 20, *Phasianus colchicus*: 4, *Phoenicopterus roseus*: 1, *Pica pica*: 266, *Pyrrhocorax graculus*: 8, *Scolopax rusticola*: 17, *Streptopelia decaocto*: 227, *Streptopelia turtur*: 18, *Sturnus vulgaris*: 2, *Turdus merula*: 39, *Turdus* *philomelos*: 21, *Upupa epops*: 4	*Accipiter brevipes*: 2, *Anas platyrhynchos*: 1, *Anser* spp.: 0, *Aquila chrysaetos* ^ *Aquila nipalensis* ^ *Aquila fasciata*: 30, Ardeidae family: 1, *Athene noctua*: 4, *Bubo bubo*: 2, *Carduelis carduelis*: 1, *Ciconia ciconia*: 1, *Columba livia*: 50, *Columba palumbus*: 3, *Corvus corax*: 46, *Corvus cornix*: 3, *Coturnix coturnix*: 5, *Cuculus canorus*: 0, *Cygnus cygnus*: 0, *Erithacus rubecula*: 1, *Falco* spp.: 55, *Fringillia coelebs*: 0, *Galerida crisata*: 0, *Garrulus glanda-rius*: 12, *Gypaetus barbatus*: 3, *Gyps* spp.: 5, *Hirundo rustica*: 59, Laridae family: 2, *Luscinia megarhynchos*: 0, Passeridae family: 45, *Pelecanus onocrotalus*: 0, *Perdix perdix*: 15, *Phasianus colchicus*: 3, *Phoenicopterus roseus*: 0, *Pica pica*: 99, *Pyrrhocorax graculus*: 4, *Scolopax rusticola*: 6, *Streptopelia decaocto*: 82, *Streptopelia turtur*: 7, *Sturnus vulgaris*: 1, *Turdus merula*: 16, *Turdus* *philomelos*: 11, *Upupa epops*: 0	*Corvus cornix*: 0.002, *Perdix perdix*: 0.013, *Hirundo rustica*: 0.022, *Anas platyrhynchos*: 0.027, *Ciconia ciconia*: 0.028, *Gypaetus barbatus*: 0.034, all others: >0.08
Hunting			
Presence of hunters in the area around the farm within a radius of 2 km of the farm	Yes: 284, no: 41	Yes: 113, no: 6	0.022
Description of hunted mammalian species	Hare: 217, wild boar: 192	Hare: 95, wild boar: 78	Hare: 0.047, wild boar: 0.39
Distance from the farm that hunting activity occurs	*X*: 1.8 ± 0.2 km	*X*: 1.2 ± 0.2 km	0.043
Production characteristics ^5^			
Average age of lambs/kids at slaughter	*X*: 50 ± 1 days	*X*: 65 ± 3 days	<0.0001
Average live bodyweight of lambs/kids at slaughter	*X*: 17.5 ± 0.2 kg	*X*: 16.6 ± 0.4 days	0.043
Average carcass weight of these at slaughter	*X*: 10.0 ± 0.1 kg	*X*: 9.4 ± 0.2 days	0.012
Local manufacturing of dairy products	Yes: 201, no: 124	Yes: 92, no: 27	0.002
Objective of local manufacturing of dairy products	Home consumption: 188, sale: 16	Home consumption: 80, sale: 13	Home consumption: 0.031, sale: 0.05
Types of dairy products in local production	Cheese: 186, yoghurt: 81, tarhana: 8, sour milk: 6, ice-cream: 5, cream: 2, pasta: 2, butter: 1, milk-rice pudding: 1	Cheese: 88, yoghurt: 29, tarhana: 1, sour milk: 2, ice-cream: 5, cream: 1, pasta: 2, butter: 3, milk-rice pudding: 1	Butter: <0.0001, all others: >0.06
Health management			
Health parameters			
The two health problems in lambs/kids considered to be of higher importance	Abscesses: 2, acidosis: 1, arthritis–encephalitis: 2, brucellosis: 0, clostridial infection: 24, coliform infections: 12, contagious ecthyma: 25, diarrhoea: 233, endoparasitic infections: 8, injuries: 2, *Listeria* infections: 1, navel infection: 1, paratuberculosis: 1, pica: 1, pneumonia: 89, selenium deficiency: 10, toxicosis: 1	Abscesses: 1, acidosis: 0, arthritis–encephalitis: 1, brucellosis: 1, clostridial infection: 12, coliform infections: 11, contagious ecthyma: 4, diarrhoea: 83, endoparasitic infections: 11, injuries: 0, *Listeria* infections: 0, navel infection: 0, paratuberculosis: 1, pica: 0, pneumonia: 27, selenium deficiency: 1, toxicosis: 1	endoparasitic infections: 0.0009, selenium deficiency: 0.008, contagious ecthyma: 0.034, all others: >0.15
Incidence rate of these two health problems in lambs/kids during the preceding season	13.0% (12.3–13.6%) ^6^	14.6% (13.7–15.6%)	0.043
The two health problems in replacement animals considered to be of higher importance	Abortion: 12, acidosis: 2, brucellosis: 1, caseous lymphadenitis: 3, cerebral coenurosis: 44, clostridial infections: 34, contagious agalactia: 3, contagious ecthyma: 1, diarrhoea: 27, endoparasitic infections: 7, enzootic intranasal tumour: 1, lameness: 4, *Listeria* infections: 1, mastitis: 12, paratuberculosis: 9, pneumonia: 53, pregnancy toxaemia: 1, vaginal prolapse: 2	Abortion: 7, acidosis: 3, brucellosis: 0, caseous lymphadenitis: 3, cerebral coenurosis: 7, clostridial infections: 7, contagious agalactia: 1, contagious ecthyma: 1, diarrhoea: 8, endoparasitic infections: 1, enzootic intranasal tumour: 2, lameness: 2, *Listeria* infections: 0, mastitis: 6, paratuberculosis: 7, pneumonia: 13, pre nancy toxaemia: 0, vaginal prolapse: 0	acidosis: 0.002, cerebral coenurosis: 0.013, caseous lymphadenitis: 0.033, all others: >0.05
The two health problems in adult animals considered to be of higher importance	Abortion: 17, acidosis: 2, brucellosis: 0, cerebral coenurosis: 5, clostridial infections: 17, contagious agalactia: 19, contagious ecthyma: 2, copper toxicosis: 2, diarrhoea: 8, endoparasitic infections: 6, enzootic intranasal tumour: 1, external myiosis: 0, hypervitaminosis A: 1, hypocalcaemia: 1, injuries: 0, lameness: 20, Lentivirus infections: 6, *Listeria* infections: 3, mange: 1, mastitis: 215, *Oestrus ovis* infestation: 1, paratuberculosis: 9, pneumonia: 57, pregnancy toxemia: 3, scrapie: 10, self-suckling: 0, sudden death: 1, toxicosis: 5, udder oedema: 1, wasting disease: 2	Abortion: 4, acidosis: 0, brucellosis: 1, cerebral coe- nurosis: 0, clostridial infections: 9, contagious agalactia: 5, contagious ecthyma: 1, copper toxicosis: 0, diarrhoea: 3, endoparasitic infections: 0, enzootic intranasal tumour: 1, external myiosis: 1, hypervitaminosis A: 1, hypocalcaemia: 0, injuries: 1, lameness: 5, Lentivirus infections: 3, *Listeria* infections: 2, mange: 0, mastitis: 51, *Oestrus ovis* infestation: 0, paratuberculosis: 23, pneumonia: 17, pregnancy toxemia: 0, scrapie: 0, self-suckling: 1, sudden death: 0, toxicosis: 3, udder oedema: 0, wasting disease: 0	mastitis: <0.0001, paratuberculosis: <0.0001, all others: >0.05
Incidence rate of these two health problems in adult animals during the preceding season	8.0% (7.2–8.7%)	6.3% (5.8–6.8%)	0.004
Incidence rate of total deaths, of any cause, in adult animals during the preceding season	5.2% (5.0–5.5%)	5.9% (5.8–6.0%)	0.023
Reasons for the visits of the veterinarians	Administration of pharmaceutical treatments: 115, feedstuff evaluation: 6, overall appraisal of flock: 163, pregnancy diagnosis: 42, vaccinations: 229	Administration of pharmaceutical treatments: 42, feedstuff evaluation: 2, overall appraisal of herd: 61, pregnancy diagnosis: 8, vaccina ions: 90	vaccinations: 0.030, pregnancy diagnosis: 0.038, all others: >0.30
Animal deaths from attacks by other animals	Yes: 118, no: 207	Yes: 70, no: 49	<0.0001
Species of animals that caused animal deaths during the preceding season	Bears: 6, crows: 1, eagles: 1, farm dogs: 23, ferrets: 1, foxes: 8, jackals: 21, wild cats: 1, wolves: 64	Bears: 3, crows: 1, eagles: 1, farm dogs: 3, ferrets: 0, foxes: 3, jackals: 20, wild cats: 2, wolves: 43	farm dogs: 0.008, jackals: 0.042, all others: >0.15
Animal deaths from natural disasters	Yes: 32, no: 293	Yes: 2, no: 97	0.014
Diseases of adult animals–mastitis			
Overall incidence rate during the preceding season	3.9% (3.8–4.0%)	2.8% (2.6–3.0%)	<0.0001
Antibiotics used for treatment	Amoxicillin: 11, ampicillin: 1, cephalosporins: 10, cloxacillin: 4, enrofloxacin: 7, florfenicol: 2, gentamicin: 4, lincomycin: 7, marbofloxacin: 6, oxytetracycline: 60, penicillin: 218, spectinomycin: 4, streptomycin: 200, tylosin: 11	Amoxicillin: 3, ampicillin: 1, cephalosporins: 1, cloxacillin: 3, enrofloxacin: 3, florfenicol: 0, gentamicin: 0, lincomycin: 3, marbofloxacin: 0, oxytetracycline: 20, penicillin: 53, spectinomycin: 2, streptomycin: 50, tylosin: 4	cloxacillin: 0.019, all others: >0.11
Diseases of adult animals–abortion			
Overall incidence rate during the preceding season	2.0% (1.9–2.1%)	2.5% (2.7–2.9%)	<0.0001
Antibiotics used for treatment	Cephalosporins: 1, lincomycin: 1, oxytetracycline: 61, penicillin: 9, streptomycin: 3, tylosin: 1	Cephalosporins: 1, lincomycin: 1, oxytetracycline: 37, penicillin: 2, streptomycin: 0, tylosin: 0	penicillin: 0.011, all others: >0.12
Diseases of adult animals–pregnancy toxemia			
Overall incidence rate during the preceding season	0.5% (0.4–0.6%)	0.2% (0.1–0.3%)	<0.0001
Treatment performed	Penicillin: 3, streptromycin: 3, ciprofloxacin: 1, dextrose: 13, oxytetracycline: 1, dexamethasone: 1, calcium: 32, propylene glycol: 11, vitamins: 12, molasses: 3, selenium: 1, trace minerals: 2, sodium: 2, nutritional change: 1	Penicillin: 0, streptromycin: 0, ciprofloxacin: 0, dextrose: 0, oxytetracycline: 1, dexamethasone: 0, calcium: 5, propylene glycol: 2 vitamins: 1, molasses: 0, selenium: 0, trace minerals: 0, sodium: 0, nutritional change: 0	dextrose: 0.024, all others: >0.07
Diseases of adult animals–lameness			
Overall incidence rate during the preceding season	2.8% (2.7–2.9%)	1.2% (1.1–1.4%)	<0.0001
Treatment performed	Lincomycin: 57, oxytetracycline: 24, cephalosporins: 16, tylosin: 4, penicillin: 2, streptomycin: 0, spectinomycin: 2, prednisolone: 0, dexamethasone: 1, quinolones: 1, non-steroidal anti-inflammatory drugs: 1, alamycin: 1, footbathing in copper/zinc solution: 33, foot pairing: 10, diesel bathing: 3, acid-based solutions: 1	Lincomycin: 12, oxytetracycline: 8, cephalosporins: 4, tylosin: 0, penicillin: 1, streptomycin: 1, spectinomycin: 0, prednisolone: 1, dexamethasone: 0, quinolones: 0, non-steroidal anti-inflammatory drugs: 0, alamycin: 0, footbathing in copper/zinc solution: 3, foot pairing: 0, diesel bathing:, acid-based solutions: 0	lincomycin: 0.0001, foot pairing: 0.001, footbathing in copper/zinc solution: 0.030, oxytetracycline: 0.048, all others: >0.12
Diseases of adult animals–mange			
Overall incidence rate during the preceding season	1.4% (1.3–1.5%)	0.1% (0.1–0.1%)	<0.0001
Diseases of adult animals–obstetrical cases			
Overall incidence rate during the preceding season	1.0% (0.9–1.1%)	0.6% (0.7–0.8%)	<0.0001
Diseases of young animals–respiratory problems			
Overall incidence rate during the preceding season	1.4% (1.3–1.5%)	1.1% (1.0–1.2%)	<0.0001
Diseases of young animals–diarrhoea			
Overall incidence rate during the preceding season	7.9% (7.8–8.0%)	10.4% (10.0–10.7%)	<0.0001
Antibiotics used for treatment	Amoxicillin: 35, ampicillin: 4, cephalosporins: 3, cloxacillin: 2, colistin: 1, enrofloxacin: 15, gentamicin: 12, lincomycin: 4, neomycin: 2, oxytetracycline: 46, penicillin: 25, spectinomycin: 13, streptomycin: 17, sulfonamides: 6, tylosin: 13	Amoxicillin: 13, ampicillin: 0, cephalosporins: 0, cloxacillin: 0, colistin: 1, enrofloxacin: 0, gentamicin: 6, lincomycin: 2, neomycin: 0, oxytetracycline: 19, penicillin: 11, spectinomycin: 5, streptomycin: 5, sulfonamides: 4, tylosin: 1	enrofloxacin: 0.014, all others: >0.16
Management practices			
Reproductive management	No hormonal control: 217, administration of melatonin: 23, administration of progestogens: 89	No hormonal control: 99, administration of melatonin: 5, administration of progestogens: 16	0.0007
Use of ultrasound for pregnancy diagnosis	Yes: 119, no: 206	Yes: 20, no: 99	<0.0001
Nutritional modifications before the lambing period	Yes: 229, no: 96	Yes: 68, no: 51	0.008
Beginning of the mating period for ewes/does	May (February–December)	June (January–December)	<0.0001
Duration of the mating period for ewe-lambs and doelings	1 (1–9) months ^7^	1 (1–6) months	0.006
Lamb/kid fostering to female animals other than their dams	Yes: 112, no: 213	Yes: 86, no: 33	<0.0001
Reasons for doing this practice	Death of dam: 39, improving nutrition of lambs: 1, inadequate milk availability by dam: 14, increased number of lambs: 112, stimulation of milk production in ewe that aborted: 31	Death of dam: 18, improving nutrition of kids: 3, inadequate milk availability by dam: 5, increased number of kids: 54, stimulation of milk production in doe that aborted: 9	increased number of lambs/kids: <0.0001, stimulation of milk production in female that aborted: 0.001, death of dam: 0.016, all others: >0.06
Age for lamb/kid removal from their dams	*X*: 50 ± 1 days	*X*: 65 ± 3 days	<0.0001
Daily number of milking sessions	2 (1–3)	2 (1–3)	0.0001
Seasonal transfer of animals to other site	Yes: 49, no: 276	Yes: 28, no: 91	0.037
Disposal of carcasses from dead animals	Burying: 183, disposal by knackers: 3, drop-off at water streams: 35, drop-off away: 52, feeding to birds: 1, feeding to dogs: 49, incineration: 28, slaughterhouse: 0	Burying: 53, disposal by knackers: 3, drop-off at water streams: 19, drop-off away: 23, feeding to birds: 0, feeding to dogs: 29, incineration: 10, slaughterhouse: 1	Feeding to dogs: 0.011, burying: 0.014, all others: >0.06
Manure management	Spread to fields: 320, disposal: 5, sale: 1	Spread to fields: 109, disposal: 7, sale: 3	<0.007
Security availability at the farm	Yes: 214, no: 111	Yes: 65, no: 54	0.03
Farm security	Alarm: 53, light wire fence: 68, personnel: 5, stoned wall: 1, strong wire fence: 93	Alarm: 13, light wire fence: 28, personnel: 1, stoned wall: 0, strong wire fence: 26	Light wire fence: 0.047, all others: >0.19
Vaccinations			
Against mastitis	Yes:126, no: 199	Yes: 34, no: 85	0.047
Against bacterial respiratory infections	Yes: 144, no: 181	Yes: 39, no: 80	0.028
Against paratuberculosis	Yes: 11, no: 314	Use: 31, no: 88	<0.0001
Antiparasitic administrations—Anthelmintic treatments to small ruminants			
Timing of administration within the annual production cycle	Before the mating season: 24, continuously: 2, final stage of pregnancy: 221, initial stage of dry period: 170, 1st–2nd month of lactation period: 89, 3rd–6th month of lactation period: 32	Before the mating season: 7, continuously: 0, final stage of pregnancy: 79, initial stage of dry period: 74, 1st–2nd month of lactation period: 33, 3rd–6th month of lactation period: 10	Initial stage of dry period: 0.026, all others: >0.29
Pharmaceutical form administered	Tablet: 221, injectable solution: 183, oral drench: 150, pour-on: 10, premix: 2	Tablet: 88, injectable solution: 71, oral drench: 47, pour-on: 9, premix: 0	Pour-on: 0.037, all others: >0.18
Antiparasitic administrations—Ectoparasiticide treatments to small ruminants			
Administration of ectoparasiticides to sheep/goats in the farm	Yes: 109, no: 216	Yes: 69, no: 50	<0.0001
Timing of administration within the annual production cycle	Before the mating season: 7, final stage of pregnancy: 60, initial stage of dry-period: 49, 1st–2nd month of lactation period: 8, 3rd–6th month of lactation period: 2	Before the mating season: 5, final stage of pregnancy: 38, initial stage of dry-period: 31, 1st–2nd month of lactation period: 8, 3rd–6th month of lactation period: 5	3rd–6th month of lactation period: 0.002, all others: >0.15
Other health management practices			
Administration of selenium to newborn animals	Yes: 228, no: 97	Yes: 67, no: 52	0.006
Shearing	Yes: 319, no: 6	Yes: 102, no: 17	<0.0001
Recording of births–maintenance of a lambing/kidding book	Yes: 182, no: 143	Yes: 53, no: 66	0.032
Disinfection of navel stumps in newborns	Yes: 213, no: 112	Yes: 65, no: 54	0.035
Tail docking in newborns	Yes: 244, no: 81	Yes: 34, no: 85	<0.0001
Maintenance of quarantine period for new animals into the farm	Yes: 211, no: 114	Yes: 61, no: 58	0.009
Vectors			
Distance of spots from farm	>50 m: 128, 50–500 m: 58, >500 m: 37	>50 m: 57, 50–500 m: 17, >500 m: 9	>50 m: 0.036, all others: >0.10
Nutrition			
Grazing practiced	Yes: 281, no: 44	Yes: 112, no: 7	0.025
Duration of grazing during the winter	*X*: 3.6 ± 0.1 months	*X*: 4.4 ± 0.2 months	0.002
Distance from farm of area grazed during the winter	*X*: 1.3 ± 0.1 km	*X*: 2.2 ± 0.2 km	0.0009
Distance from farm of area grazed during the summer	*X*: 1.6 ± 0.1 km	*X*: 2.4 ± 0.2 km	0.004
Type of graze area	Forest: 18, hay: 4, meadow: 204, olive trees: 2, scrub pasture: 83, wetland: 16	Forest: 23, hay: 5, meadow: 59, olive trees: 0, scrub pasture: 58, wetland: 5	Meadow, scrub pasture, forest: <0.0001, all others: >0.26
Common grazing for sheep and goats	Yes: 48, no: 277	Yes: 40, no: 79	<0.0001
Provision of hay as fodder to animals	Yes: 324, no: 1	Yes: 116, no: 3	0.029
Total quantity of hay consumed during the preceding season	*X*: 84 ± 5 tonnes	*X*: 46 ± 7 tonnes	0.0003
Plants included in hay consumed by animals	*Avena sativa*: 25, *Euphorbia pulcherrima*: 1, *Hordeum vulgare*: 22, *Lolium*: 15, *Medicago sativa*: 10, *Pisum sati-vum*: 7, *Polygonum aviculare*: 2, *Trifolium*: 295, *Triticum*: 5, *Vicia*: 25	*Avena sativa*: 21, *Euphorbia pulcherrima*: 0, *Hordeum vulgare*: 8, *Lolium*: 1, *Medi* *cago sativa*: 2, *Pisum sati-vum*: 2, *Polygonum aviculare*: 0, *Trifolium*: 106, *Triticum*: 0, *Vicia*: 7	*Lolium*, *Medicago sativa*, *Pisum sativum*, *Triticum*: <0.0001, all others: >0.09
Origin of hay	Own production: 193, purchase: 206	Own production: 55, purchase: 81	Own production: 0.005, purchase: 0.11
Provision of straw to animals	Yes: 258, no: 67	Yes: 78, no: 41	0.003
Provision of finished feed to animals	Yes: 304, no: 21	Yes: 103, no: 16	0.018
Provision of finished feed (concentrate) to animals throughout the year	Yes: 304, no: 21	Yes: 103, no: 16	0.018
Provision of finished feed (concentrate) to young animals	Yes: 255, no: 70	Yes: 79, no: 40	0.009
Finished feed (concentrate) type provided to young animals	Flakes: 0, mash: 102, pellets: 82, small pellets: 80	Flakes: 0, mash: 26, pellets: 22, small pellets: 35	Small pellets: 0.017, all others: >0.12
Raw materials used by the farm in the diets	Barley: 227, bran: 185, cottonseed meal: 84, maize: 294, soyabean meal: 201: sunflower meal: 78, wheat: 104	Barley: 86, bran: 66, cottonseed meal: 42, maize: 110, soyabean meal: 68: sunflower meal: 28, wheat: 42	cottonseed meal: 0.025, all others: >0.18
Raw materials purchased by the farm for use in the diets	Barley: 205, bran: 196, cottonseed meal: 100, maize: 258, soyabean meal: 215, sunflower meal: 87, wheat: 87	Barley: 84, bran: 68, cottonseed meal: 49, maize: 105, soyabean meal: 68, sunflower meal: 29, wheat: 41	maize: 0.016, cottonseed meal: 0.020, soyabean meal: 0.040, all others: >0.05
Feed change in animals	Abrupt: 3, progressive: 314	Abrupt: 2, progressive: 114	<0.0001
Application of hydroponics cultivations	Yes: 4, no: 321	Yes: 1, no: 118	<0.0001
Use of laboratory examinations for quality testing of feeds and raw material	Yes: 79, no: 246	Yes: 19, no: 100	<0.0001
Laboratory examinations used	Chemical analysis: 37, microbiological examination: 4, mycotoxin detection: 39, residues analysis: 3	Chemical analysis: 7, microbiological examination: 1, mycotoxin detection: 8, residues analysis: 2	Residues analysis: 0.009, all others: >0.21
Use of laboratory examinations for quality testing of water	Yes: 52, no: 273	Yes: 13, no: 106	<0.0001
Person responsible for nutritional management	Farmer themselves: 198, veterinarian: 96, animal scientist: 81, other farmer: 0	Farmer themselves: 94, veterinarian: 28, animal scientist: 22, other farmer: 0	Farmer themselves: 0.0002, all others: >0.07
Human Resources			
Farmer			
Professional education	Yes: 54, no: 171	Yes: 12, no: 107	0.002
Daily period of presence in the farm	*X*: 11.5 ± 0.2 h	*X*: 12.3 ± 0.3 h	0.047
Public health			
Personal opinion regarding occurrence of transmission of diseases from animals to the farmer or members of the family	Yes: 44, no: 281	Yes: 27, no: 92	0.020

^1^ *p*-value for comparison between sheep flocks and goat herds; ^2^ Management system classified according to the system of the European Food Safety Authority [24]; ^3^ Figures present frequency (*n*) for each category within the variable; ^4^ Figures present mean ± standard error of the mean (*X* ± σ_M_.); ^5^ Figures present frequency (*n*) for each category within the variable; ^6^ Figures present average (95% confidence interval) value; ^7^ Figures present median (minimum–maximum) value.

## Appendix B

**Table A2:** Differences observed in parameters (*n* = 7) between sheep flocks and goat herds in the results obtained during field and laboratory examinations during the mapping of 444 small ruminant farms in a countrywide investigation in Greece.

	Sheep Flocks (*n* = 325)	Goat Herds (*n* = 199)	*p*-Value ^1^
Clinical Examinations of Animals at the Farms			
Body condition scoring	*X*: 2.38 ± 0.02 (scale: 0–5) ^2^	*X*: 2.54 ± 0.03 (scale: 0–5) ^3^	<0.0001
Laboratory Examinations in Bulk-Tank Milk			
Somatic cell counting			
Somatic cell counts	0.488 × 10^6^(0.451 × 10^6^–0.529 × 10^6^) cells mL^−1 3^	0.838 × 10^6^ (0.759 × 10^6^–0.933 × 10^6^) cells mL^−1^	<0.0001
Microbiological examinations			
Total bacterial counts	398 × 10^3^ (331 × 10^3^–479 × 10^3^) cfu mL^−1^	581 × 10^3^ (447 × 10^3^–741 × 10^3^) cfu mL^−1^	<0.0001
Resistance of staphylococci to ampicillin, azithromycin, cefoxitin, ciprofloxacin, clarithromycin, clindamycin, erythromycin, fosfomycin, fucidic acid, gentamicin, moxifloxacin, mupirocin, oxaxillin, penicillin, rifampicin, teicoplanin, tetracycline, tobramycin, trimethoprim-sulfamethoxazole	Yes: 79, 0, 0, 2, 0, 41, 21, 31, 14, 2, 1, 1, 27, 79, 1, 0, 28, 2, 2, respectively, no: 153, 232, 232, 230, 232, 191, 211, 201, 218, 230, 231, 231, 205, 153, 231, 232, 204, 230, 230, respectively ^4^	Yes: 33, 0, 0, 0, 0, 19, 16, 22, 3, 0, 0, 0, 6, 33, 0, 1, 12, 1, 0, respectively, no: 47, 80, 80, 80, 80, 61, 64, 58, 77, 80, 80, 80, 74, 47, 80, 79, 68, 79, 80, respectively	Fosfomycin: 0.002, erythromycin: 0.040, all others: >0.11
Composition analysis			
Fat content	*X*: 6.16% ± 0.05%	*X*: 4.77% ± 0.44%	0.0005
Protein content	*X*: 4.43% ± 0.01%	*X*: 3.23% ± 0.30%	0.0008
Lactose content	*X*: 4.21% ± 0.02%	*X*: 4.74% ± 0.03%	<0.0001

^1^ *p*-value for comparison between sheep flocks and goat herds; ^2^ Figures present mean ± standard error of the mean (*X* ± σ_M_.); ^3^ Figures present mean (95% confidence interval); ^4^ Figures present frequency (*n*) for each category within the variable.

## References

[1] Hellenic Statistical Authority Farm Structure Surveys. https://www.statistics.gr.

[2] Pulina G., Milán M.J., Lavín M.P., Theodoridis A., Morin E., Capote J., Thomas D.L., Francesconi A.H.D., Caja G. (2018). Invited review: Current production trends, farm structures, and economics of the dairy sheep and goat sectors. J. Dairy Sci..

[3] Ministry of Agricultural Development & Food (2018). Greek Agriculture—Animal Production.

[4] Hellenic Agricultural Organisation—Demeter Deliveries of Ovine and Caprine Milk by Region and Regional Authority—Calendar Year 2022. https://www.elgo.gr/images/ELOGAK_files/Statistics/ELGO_STATS/1.ΕΛΓO_STATS_ΠAΡAΔ_ΠOΣ__ΠAΡAΓ_ΠΡOΒΕΙOΥ_ΓΙΔΙΝO_AΝA_ΝOΜO__ΜHΝA_2022.pdf.

[5] Hadjigeorgiou I., Vallerand F., Tsimpoukas K., Zervas G. (2002). The socio-economics of sheep and goat farming in Greece and the implications for future rural development. Opt. Méditerran. B Etud. Recherch..

[6] Hellenic Agricultural Organisation—Demeter Deliveries of Bovine and Bubaline Milk by Region and Regional Authority—Calendar Year 2022. https://www.elgo.gr/images/ELOGAK_files/Statistics/ELGO_STATS/1.ΕΛΓO_STATS_ΠAΡAΔ_ΠOΣ__ΠAΡAΓ_AΓΕΛAΔΙΝO_ΒOΥΒAΛΙΣΙO_AΝA_ΝOΜO__ΜHΝA_2022.pdf.

[7] Kizos T., Plieninger T., Schaich H. (2013). “Instead of 40 sheep there are 400”: Traditional grazing practices and landscape change in Western Lesvos, Greece. Landsc. Res..

[8] Manolopoulou E., Aktypis A., Matara C., Tsiomi P., Konstantinou E., Mountzouris K., Klonaris S., Tsakalidou E. (2018). An overview of sheep farming features and management practices in the region of south western Peloponnese and how they reflect on milk microbial load. J. Hell. Vet. Med. Soc..

[9] Perucho L., Hadjigeorgiou I., Lauvie A., Moulin C.H., Paoli J.C., Ligda C. (2019). Challenges for local breed management in Mediterranean dairy sheep farming: Insights from Central Greece. Trop. Anim. Health Prod..

[10] Kominakis A.P., Papavasiliou D., Rogdakis E. (2009). Relationships among udder characteristics, milk yield and, non-yield traits in Frizarta dairy sheep. Small Rumin. Res..

[11] Gelasakis A.I., Valergakis G.E., Arsenos G., Banos G. (2012). Description and typology of intensive Chios dairy sheep farms in Greece. J. Dairy Sci..

[12] Lianou D.T., Chatziprodromidou I.P., Vasileiou N.G.C., Michael C.K., Mavrogianni V.S., Politis A.P., Kordalis N.G., Billinis C., Giannakopoulos A., Papadopoulos E. (2020). A detailed questionnaire for the evaluation of health management in dairy sheep and goats. Animals.

[13] Lianou D.T., Michael C.K., Gougoulis D.A., Cripps P.J., Vasileiou N.G.C., Solomakos N., Petinaki E., Katsafadou A.I., Angelidou E., Arsenopoulos K.V. (2022). High milk somatic cell counts and increased *Teladorsagia* burdens overshadow non-infection-related factors as predictors of fat and protein content of bulk-tank raw milk in sheep and goat farms. Foods.

[14] Lianou D.T., Arsenopoulos K.V., Michael C.K., Papadopoulos E., Fthenakis G.C. (2022). Protozoan parasites in adult small ruminants and potential predictors for their presence in faecal samples. Microorganisms.

[15] Martin W.B., Aitken I.A., Martin W.B., Aitken I.A. (2000). Appendix C. Diseases of Sheep.

[16] Laird D.T., Gambrel-Lenarz S.A., Scher F.M., Graham T.E., Reddy R., Wehr H.M., Frank J.F. (2004). Microbiological Count Methods. Standard Methods for the Examination of Dairy Products.

[17] Barrow G.I., Feltham R.K.A. (1993). Manual for the Identification of Medical Bacteria.

[18] Euzeby J.P. (1997). List of bacterial names with standing in nomenclature: A folder available on the Internet. Int. J. Syst. Bacteriol..

[19] (2017). Microbiology of the Food Chain—Horizontal Method for the Detection and Enumeration of Listeria Monocytogenes and of *Listeria* spp.—Part 1: Detection Method.

[20] Vasileiou N.G.C., Chatzopoulos D.C., Gougoulis D.A., Sarrou S., Katsafadou A.I., Spyrou V., Mavrogianni V.S., Petinaki E., Fthenakis G.C. (2018). Slime-producing staphylococci as causal agents of subclinical mastitis in sheep. Vet. Microbiol..

[21] Rinaldi L., Levecke B., Bosco A., Ianniello D., Pepe P., Charlier J., Cringoli G., Vercruysse J. (2014). Comparison of individual and pooled faecal samples in sheep for the assessment of gastrointestinal strongyle infection intensity and anthelmintic drug efficacy using McMaster and Mini-FLOTAC. Vet. Parasitol..

[22] Henriksen S.A., Pohlenz J.F.L. (1981). Staining of cryptosporidia by a modified Ziehl-Neelsen technique. Acta Vet. Scand..

[23] Lianou D.T., Michael C.K., Vasileiou N.G.C., Petinaki E., Cripps P.J., Tsilipounidaki K., Katsafadou A.I., Politis A.P., Kor-dalis N.G., Ioannidi K.S. (2021). Extensive countrywide field investigation of somatic cell counts and total bacterial counts in bulk-tank raw milk in sheep flocks in Greece. Foods.

[24] European Food Safety Authority (2014). Scientific opinion on the welfare risks related to the farming of sheep for wool, meat and milk production. EFSA J..

[25] Lianou D.T., Vasileiou N.G.C., Michael C.K., Valasi I., Mavrogianni V.S., Caroprese M., Fthenakis G.C. (2022). Patterns of reproductive management in sheep and goat farms in Greece. Animals.

[26] Lianou D.T., Fthenakis G.C. (2022). Use of antibiotics against bacterial infections on dairy sheep and goat farms: Patterns of usage and associations with health management and human resources. Antibiotics.

[27] Lianou D.T., Michael C.K., Petinaki E., Mavrogianni V.S., Fthenakis G.C. (2022). Administration of vaccines in dairy sheep and goat farms: Patterns of vaccination, associations with health and production parameters, predictors. Vaccines.

[28] Lianou D.T., Fthenakis G.C. (2022). Scientometrics study of research output on sheep and goats from Greece. Animals.

[29] Lianou D.T., Fthenakis G.C. (2021). Dairy sheep and goat farmers: Socio-demographic characteristics and their associations with health management and performance on farms. Land.

[30] Anyfantakis E.M. (2004). Cheese Production, Chemistry, Physicochemistry, Microbiology.

[31] Papadopoulos A. (2009). Changes of Characteristics of Mechanically Produced Ovine Milk from the Udder to the Bulk-Tank. Master’s Thesis.

