# Supplementary material for: Data on Mapping 444 Dairy Small Ruminant Farms during a Countrywide Investigation Performed in Greece
Source: Animals (Basel). 2023 Jun 20;13(12):2044. doi: 10.3390/ani13122044 (PMC10295351; doi:10.3390/ani13122044)
Supplement: Supplementary file 1 [file animals-13-02044-s001.zip › animals-2438635-supplementary.pdf]

Data of Mapping 444 Dairy Small Ruminant Farms during a Countrywide Investigation Performed in Greece

Daphne T. Lianou, Charalambia K. Michael and George C. Fthenakis

Table S1. Geographical areas of Greece (n = 4), in which administrative regions and regional units of the country were clustered, for characterizing location of 444 small ruminant farms in a countrywide investigation in Greece.

**Central**
Region of Central Greece
Region of Thessaly
From the region of Epirus: regional unit of Arta and regional unit of Preveza
From the region of Western Greece: regional unit of Aetolia-Acarnania
**Islands**
Region of Crete
Region of Ionian islands
Region of North Aegean
Region of South Aegean
**North**
Region of Central Macedonia
Region of Eastern Maecedonia and Thrace
Region of Western Macedonia
From the region of Epirus: regional unit of Ioannina and regional unit of Thesprotia
**South**
Region of Attica
Region of Peloponnese
Region of Western Macedonia
From the region of Western Greece: regional unit of Achaea and regional unit of Elis

Figure S1. Map of Greece indicating the geographic areas of the country (*n* = 4), in which administrative regions and regional units of the country were clustered, for characterizing location of 444 small ruminant farms in a countrywide investigation in Greece (blue: Central, green: Islands, yellow: North, red: South).

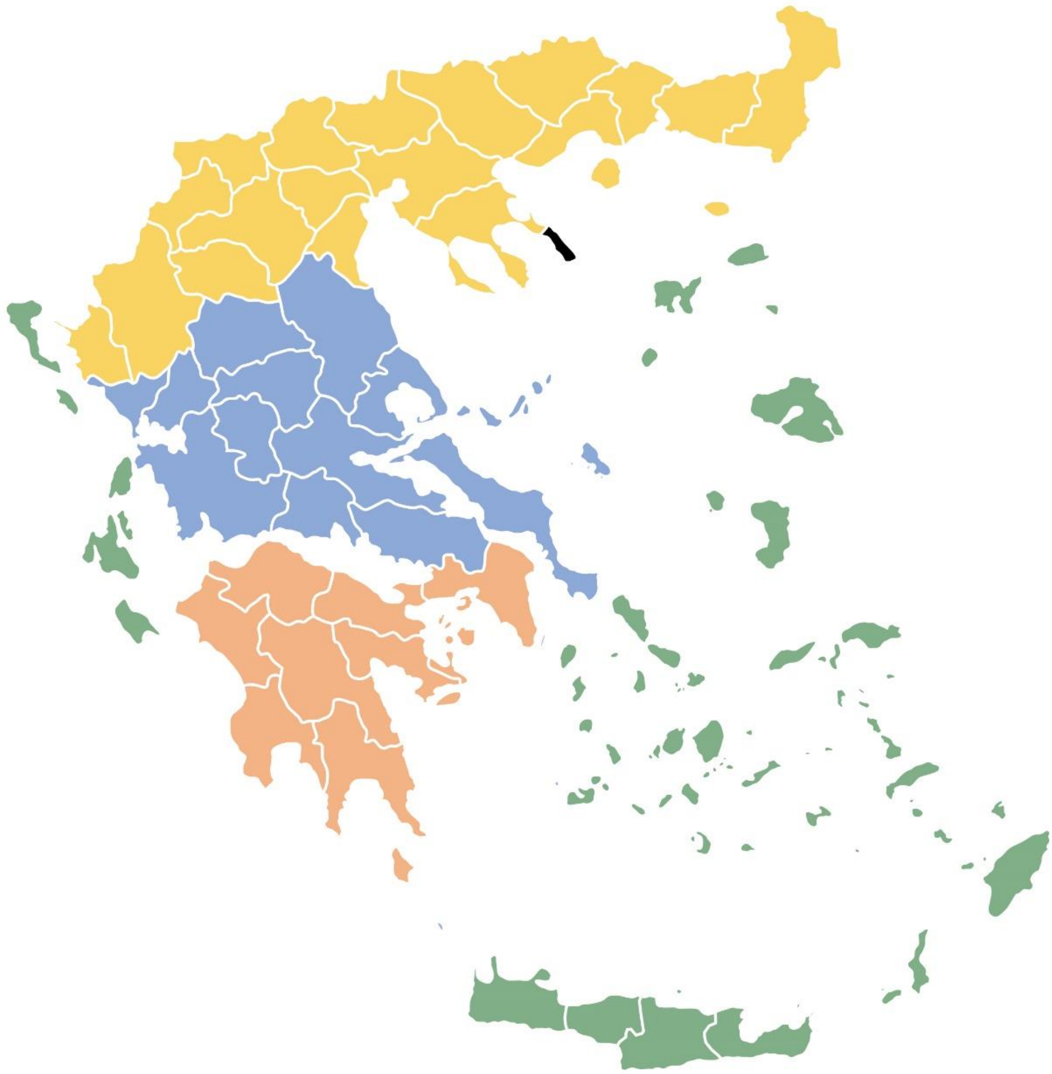

Table S2. Cumulative findings of all data collected during mapping 444 small ruminant farms in a countrywide investigation in Greece, classified according to animal species in the farms.

	SHEEP FLOCKS<br>*n* = 325	GOAT HERDS<br>*n* = 199	*p*-value [1]
**GENERAL DETAILS**			
Management system [2]	Intensive: 44, semi-intensive: 140, semi-extensive: 116, extensive: 25	Intensive: 9, semi-intensive: 29, semi-extensive: 61, extensive: 20	< 0.0001 [3]
**INFRASTRUCTURE**			
**General information**			
Year of the initial establishment of the farm	$\bar{X}$: year 1981 ± 1 year [4]	$\bar{X}$: year 1977 ± 1 year	0.09
Year of establishment at the present location	$\bar{X}$: year 2000 ± 1 year	$\bar{X}$: year 2000 ± 1 year	0.93
Availability of a main building for animals	Yes: 318, no: 7 [5]	Yes: 117, no: 2	0.75
Availability of a dedicated building for lambs / kids	Yes: 243, no: 82	Yes: 86, no: 33	0.59
Availability of a separate lambing / kidding area	Yes: 173, no: 152	Yes: 58, no: 61	0.40
Is the separate lambing / kidding area permanent	Yes: 84: no: 89	Yes: 31, no: 27	0.52
Accessory building(s) for animals	Yes: 222, no: 103	Yes: 93, no: 26	0.043
Availability of sheds	Yes: 233, no: 92	Yes: 96, no: 23	0.06
Number of available sheds	$\bar{X}$: 2 ± 0.1 sheds	$\bar{X}$: 2 ± 0.1 sheds	0.50
Availability of sheds for storage of concentrate feedstuffs	Yes: 277, no: 48	Yes: 106, no: 13	0.30
Availability of sheds for storage of roughage	Yes: 281, no: 44	Yes: 100, no: 19	0.52
Availability of sheds for storage of silage	Yes: 64, no: 261	Yes: 16, no: 103	0.13
Availability of a milking parlour	Yes: 255, no: 70	Yes: 66, no: 53	< 0.0001
Availability of a waiting area before the milking parlour	Yes: 226, no: 29	Yes: 64, no: 2	0.041
Availability of personnel areas	Yes: 179, no: 145	Yes: 40, no: 79	0.009
Availability of area for veterinary works	Yes: 63, no: 262	Yes: 14, no: 105	0.06
Availability of an office	Yes: 128, no: 197	Yes: 34, no: 85	0.036
Availability of a lavatory	Yes: 143, no: 182	Yes: 38, no: 81	0.022
Availability of footbath	Yes: 45, no: 280	Yes: 10, no: 109	0.005
Availability of isolation facilities for animals	Yes: 247, no: 78	Yes: 77, no: 42	0.018
Availability of access road to the farm	Yes: 251, no: 74	Yes: 78, no: 41	0.013
Availability of electricity:	Yes: 293, no: 32	Yes: 104, no: 15	0.40
Electricity source	National network: 253, diesel generator: 48, own solar cells: 0	National network: 72, diesel generator: 29, own solar cells: 3	0.0002
Proximity to industrial sites	Yes: 54, no: 271	Yes: 12, no: 107	0.09
Distance to industrial sites	$\bar{X}$: 1.9 ± 0.1 km	$\bar{X}$: 2.1 ± 0.2 km	0.72
Type of industrial sites near the farm	Food processing: 27, Construction-related: 11, Other light industries: 8, Heavy industries: 9	Food processing: 5, Construction-related: 3, Other light industries: 1, Heavy industries: 3	0.90
Availability of waste processing facility	Yes: 3, no: 322	Yes: 1, no: 118	0.93
Availability of a ditch at the main entrance	Yes: 0, no: 325	Yes: 0, no: 119	n/a
Recording of details of incoming vehicles	Yes: 19, no: 306	Yes: 8, no: 111	0.73
**Main building**			
Material of the walls	Cinder blocks: 130, tin: 99, bricks: 69, panels: 44, wood: 21, cement: 7, stone: 5, canvas: 4, nylon: 4, plaster: 1, plastic: 1	Cinder blocks: 57, tin: 38, bricks: 19, panels: 13, wood: 8, cement: 2, stone: 7, canvas: 0, nylon: 1, plaster: 0, plastic: 0	stone: 0.014 all others: > 0.08
Material of the roof	Tin: 213, panels: 82, tiles: 15, wood: 15, canvas: 7, cinder blocks: 6, bricks: 2, cement: 0	Tin: 81, panels: 22, tiles: 5, wood: 7, canvas: 2, cinder blocks: 3, bricks: 0, cement: 2	all: > 0.25

Dimensions of the building	Length: $\bar{X}$: 9.4 ± 0.2 m<br>Width: $\bar{X}$: 5.0 ± 0.1 m<br>Height: $\bar{X}$: 3.7 ± 0.1 m<br>Total volume:<br>$\bar{X}$: 146 ± 8 m^3	Length: $\bar{X}$: 10.0 ± 0.4 m<br>Width: $\bar{X}$: 5.1 ± 0.2 m<br>Height: $\bar{X}$: 3.8 ± 0.1 m<br>Total volume:<br>$\bar{X}$: 126 ± 18 m^3	> 0.14
Openings in the walls	Yes: 314, no: 4	Yes: 115, no: 2	0.72
Number of openings in the walls	All around: 174, remaining: $\bar{X}$: 10 ± 0.5 openings	All around: 60, remaining: $\bar{X}$: 12 ± 1.4 openings	0.55
Opening in the roof	Yes: 234, no: 84	Yes: 35, no: 82	< 0.0001
Orientation of the building	$\bar{X}$: 136 ± 6 °	$\bar{X}$: 131 ± 8 °	0.76
Material of the floor	Soil: 293, concrete: 29, stone: 16, slatted metal: 2, slatted wood: 2, slatted plastic: 1, wood: 1	Soil: 105, concrete: 12, stone: 1, slatted metal: 1, slatted wood: 2, slatted plastic: 0, wood: 2,	stone: 0.0002, wood: 0.015, all others: > 0.22
Availability of straw bedding	Yes: 268, no: 60	Yes: 76, no: 41	0.0002
Annual frequency of removal / clean-up of the straw bedding	$\bar{X}$: 3 ± 0.5 occasions	$\bar{X}$: 5 ± 0.5 occasions	0.003
Availability of ventilators	Yes: 47, no: 281	Yes: 8, no: 109	0.035
Availability of artificial lighting	Yes: 278, no: 40	Yes: 83, no: 34	< 0.0001
**Building for lambs / kids**			
Availability of milk replacer facilities	Yes: 22, no: 303	Yes: 1, no: 118	0.013
Availability of milk heating facilities	Yes: 20, no: 2	Yes: 1, no: 0	0.75
Number of plastic teats available	$\bar{X}$: 22 ± 2 teats	$\bar{X}$: 23 teats	0.97
Openings in the walls	Yes: 28, no: 215	Yes: 1, no: 85	0.004
Opening in the roof	Yes: 28, no: 215	Yes: 1, no: 85	0.004
Daily frequency of administration of milk replacer to lambs / kids	$\bar{X}$: 2 ± 0.1 occasions	$\bar{X}$: 2 occasions	0.88
Grouping of lambs / kids for administration of the milk replacer	Yes: 19, no: 3	Yes: 1, no: 0	0.69
Criteria for grouping of lambs / kids for administration of the milk replacer	Age: 19, weight: 10, milk production of dam: 3	Age: 1, weigh: 1, milk production of dam: 0	> 0.55
**Milking parlour**			
Year of initial establishment	$\bar{X}$: year 2010 ± 0.5 year	$\bar{X}$: year 2012 ± 0.5 year	0.030
Year of most recent renovation	$\bar{X}$: year 2014 ± 0.5 year	$\bar{X}$: year 2015 ± 0.5 year	0.42
Dimensions of the parlour	Length: $\bar{X}$: 9.5 ± 0.2 m<br>Width: $\bar{X}$: 5.0 ± 0.1 m<br>Height: $\bar{X}$: 3.7 ± 0.1 m<br>Total volume:<br>$\bar{X}$: 201 ± 9 m^3	Length: $\bar{X}$: 9.9 ± 0.5 m<br>Width: $\bar{X}$: 4.8 ± 0.2 m<br>Height: $\bar{X}$: 3.6 ± 0.1 m<br>Total volume:<br>$\bar{X}$: 197 ± 21 m^3	> 0.23
Material of the floor	Concrete: 135, tiles: 51, soil: 39, wood: 19, plastic: 3, stone: 2, tin: 2, canvas: 1, metal: 1, tar: 1	Concrete: 35, tiles: 9, soil: 14, wood: 8, plastic: 0, stone: 0, tin: 0, canvas: 0, metal: 0, tar: 0	0.72
Type of milking parlour	Non-mobile: 216, mobile: 39	Non-mobile: 58, mobile: 6	0.22
Type of milking parlour	Linear one-sided: 152, linear parallel: 91, circular: 10, fishbone: 1, other: 1	Linear one-sided: 41, linear parallel: 21, circular: 4, fishbone: 0, other: 0	0.85
Number of animal positions in the parlour	$\bar{X}$: 25 ± 1 positions	$\bar{X}$: 25 ± 2 positions	0.65
Number of available milking units	$\bar{X}$: 15 ± 0.5 units	$\bar{X}$: 16 ±1.5 units	0.42
Provision of feed during milking	Yes: 230, no: 25	Yes: 58, no: 8	0.58
Availability of facilities for milk yield measurement	Yes: 14, no: 242	Yes: 3, no: 63	0.76
Type of facilities for milk yield measurement	Individual animal level: 13, farm level: 1	Individual animal level: 3, farm level: 0	0.63
Availability of milk quality indicators	Yes: 0, no: 255	Yes: 0, no: 66	n/a
Availability of milk flow indicators	Yes: 3, no: 252	Yes: 1, no: 65	0.83
System pulsation rate	$\bar{X}$: 145 ± 1.5 p. min^{-1}	$\bar{X}$: 141 ± 2.0 p. min^{-1}	0.22

System pressure	$\bar{X}$: 40 ± 0.5 kPa	$\bar{X}$: 39 ± 0.5 kPa	0.22
Type of flow line	Low: 177, high: 54, other: 23	Low: 40, high: 16, other: 10	0.42
Frequency of check-ups of the system by farmer	Daily: 220, weekly: 13, monthly: 6, bi-monthly: 3, quarterly: 2, six-monthly: 2, yearly: 2, never: 7	Daily: 54, weekly: 3, monthly: 2, bi-monthly: 1, quarterly: 1, six-monthly: 0, yearly: 2, never: 3	0.80
Annual frequency of check-ups of the system by technicians	Once: 123, twice: 54, more than twice: 42	Once: 24, twice: 15, more than twice: 11	0.57
System check-ups performed by technicians	Yes: 219, no: 36	Yes: 50, no: 16	0.047
Type of system check-ups performed by technicians	Full: 214, partial: 5	Full: 50, partial: 0	0.28
Water cleaning of parlour after the milking sessions	Yes: 243, no: 12	Yes: 64, no: 2	0.55
Temperature of cleaning water	$\bar{X}$: 70 ± 0.5 °C	$\bar{X}$: 70 ± 1.0 °C	0.96
Use of detergent for parlour cleaning after the milking session	Yes: 252, no: 3	Yes: 65, no: 1	0.83
Criteria for changing teatcups	Time: 254, no. of milkings: 1	Time: 66, no. of milkings: 0	0.61
Annual frequency of changing teatcups	Once: 130, less than once: 125	Once: 29, less than once: 37	0.31
Availability of a milk tank	Yes: 307, no: 18	Yes: 103, no: 16	0.006
Availability of a mixer in the milk tank	Yes: 306, no: 1	Yes: 101, no: 2	0.10
Temperature in the milk tank	$\bar{X}$: 3.7 ± 0.1 °C	$\bar{X}$: 3.6 ± 0.1 °C	0.70
Frequency of milk collection by or delivery to the dairy processing plant	Daily: 82, every two days: 226, every three days: 17, every four days: 0	Daily: 36, every two days: 77, every three days: 4, every four days: 2	0.06
**Equipment**			
Availability of a crate	Yes: 41, no: 284	Yes: 9, no: 110	0.14
Availability of scales	Yes: 222, no: 103	Yes: 76, no: 43	0.38
Scale type available	Large: 114, portable: 140	Large: 28, portable: 59	< 0.015
Availability of a roller crusher	Yes: 163, no: 162	Yes: 40, no: 79	0.002
Availability of a feed mill	Yes: 134, no: 191	Yes: 34, no: 85	0.015
Availability of an automated feeding system	Yes: 61, no: 264	Yes: 22, no: 97	0.95
Availability of a silage distributor	Yes: 32, no: 293	Yes: 10, no: 109	0.65
Availability of a straw cutter	Yes: 41, no: 284	Yes: 12, no: 107	0.47
Availability of automatic water filling system in troughs	Yes: 167, no: 158	Yes: 40, no: 79	0.0009
Availability of system for waste removal	Yes: 5, no: 320	Yes: 2, no: 117	0.92
Availability of bathing tank	Yes: 4, no: 321	Yes: 1, no: 118	0.73
Availability of a generator	Yes: 165, no: 160	Yes: 59, no: 60	0.82
Availability of a tractor	Yes: 235, no: 90	Yes: 70, no: 49	0.007
Availability of a truck	Yes: 63, no: 262	Yes: 23, no: 96	0.99
Availability of a pick-up	Yes: 285, no: 40	Yes: 104, no: 15	0.93
Use of animal identification	Yes: 322, No: 3	Yes: 117, No: 2	0.50
Animal identification system used	Ear-tags: 311, intra-ruminal boli: 9, necklace: 2	Ear-tags: 115, intra-ruminal boli: 2, necklace: 0	0.56
Availability of automatic animal identification system	Yes: 2, no: 323	Yes: 0, no: 119	0.39
Availability of animal location identifiers	Yes: 1, no: 324	Yes: 0, no: 119	0.54
Means of feed administration	Automated feeding belt: 7, bulk feeding: 57, troughs: 261	Automated feeding belt: 0, bulk feeding: 13, troughs: 106	0.06
Total number of feed troughs available	$\bar{X}$: 28 ± 2 troughs	$\bar{X}$: 26 ± 3 troughs	0.71
Type of feed troughs available	Wood: 101, metal: 193, concrete: 1, plastic: 1	Wood: 41, metal: 75 concrete: 0, plastic: 2	> 0.06
Total number of drinking points available	$\bar{X}$: 10 ± 0.5 points	$\bar{X}$: 9 ± 1.0 points	0.46
Availability of refrigerators	Yes: 216, no: 109	Yes: 69, no: 50	0.10
Description of available refrigerators	Domestic type: 205, professional type: 21	Domestic type: 65, professional type: 7	> 0.18
Recording of environmental conditions	Yes: 61, no: 264	Yes: 16, no: 103	0.19
Available facilities for recording of environmental conditions	Thermometres: 61, hygrometres: 32	Thermometres: 16, hygrometers: 8	> 0.43
Practicing sharing of equipment with other farms	Yes: 14, no: 311	Yes: 4, no: 115	0.65

**Land**			
Grazing practiced	Yes: 281, no: 44	Yes: 112, no: 7	0.025
Total grazing land	$\bar{X}$: 510 ± 50 acres	$\bar{X}$: 1322 ± 390 acres	0.002
Ownership of the grazing land	Farmer's: 225, other private: 175, public: 103	Farmer's: 86, other private: 55, public: 58	public: 0.003, other private: 0.01, farmer's: > 0.20
Private grazing land	$\bar{X}$: 152 ± 18 acres	$\bar{X}$: 343 ± 113 acres	0.018
Irrigation of the private grazing land	Yes: 123, no: 115	Yes: 27, no: 65	0.0003
Grazing of cultivated land	Yes: 199, no: 82	Yes: 73, no: 39	0.27
Cultivated grazing land	$\bar{X}$: 127 ± 10 acres	$\bar{X}$: 117 ± 13 acres	0.62
Plant types available in the grazing land	Oat: 151, clover: 55, ryegrass: 53, barley: 50, vetch: 31, corn: 13, sorghum: 10, alfalfa: 6, rye: 4, olive trees: 3, spurges: 2, poppies: 1, wild grass: 1, wheat: 0	Oat: 44, clover: 15, ryegrass: 16, barley: 28, vetch: 8, corn: 8, sorghum: 3, alfalfa: 2, rye: 3, olive trees: 0, spurges: 0, poppies: 0, wild grass: 1, wheat: 7	oat: 0.005, all others: > 0.05
Use of hydroponic facilities	Yes: 2, no: 323	Yes: 1, no: 118	0.80
**ANIMALS**			
**Small ruminants**			
No. of female animals	$\bar{X}$: 325 ± 13 ewes	$\bar{X}$: 237 ± 20 does	0.0006
No. of male animals	$\bar{X}$: 15 ± 0.5 rams	$\bar{X}$: 16 ± 1.5 bucks	0.47
Breed of female animals	Assaf: 30, Awassi: 1, Boutsko: 2, Chios: 44, Crossbreds: 43, Friesarta: 12, Friesian: 13, Karagouniko: 5, Kefallinia: 1, Lacaune: 95, 'Local': 55, Mytilini: 18, Sfakia: 6	Alpine: 9, Crossbreds: 18, Damascus: 18, Kefallinia: 1, Indigenous Greek (*Capra prisca*): 50, Murciano-Granadina: 13, Saanen: 5, Skopelos: 5	n/a
Breed of male animals	Assaf: 27, Awassi: 1, Boutsko: 1, Chios: 36, Crossbreds: 93, Friesarta: 9, Friesian: 7, Karagouniko: 5, Kefallinia: 0, Lacaune: 67, 'Local': 68, Mytilini: 5, Sfakia: 6	Alpine: 11, Crossbreds: 33, Damascus: 12, Kefallinia: 1, Indigenous Greek (*Capra prisca*): 48, Murciano-Granadina: 9, Saanen: 2, Sfakia: 1, Skopelos: 2	n/a
Average age of culling ewes / does	$\bar{X}$: 5.9 ± 0.1 years	$\bar{X}$: 6.9 ± 0.1 years	< 0.0001
Average age of culling rams / bucks	$\bar{X}$: 4.4 ± 0.2 years	$\bar{X}$: 4.9 ± 0.2 years	0.009
Average annual replacement rate of ewes / does	$\bar{X}$: 17.0% ± 0.2%	$\bar{X}$: 14.5% ± 0.3%	< 0.0001
Average annual replacement rate of rams / bucks	$\bar{X}$: 22.5% ± 1.0%	$\bar{X}$: 20.5% ± 1.0%	0.009
Source of replacement animals	Own animals: 165, purchase: 24, both sources: 136	Own animals: 77, purchase: 6, both sources: 35	0.026
Criteria for selection of own animals as replacements	Dam milk production: 254, milkability: 65, general animal morphology: 54, introduction of new pedigree: 8, dam prolificacy: 2	Dam milk production: 98, milkability: 21, general animal morphology: 27, introduction of new pedigree: 1, dam prolificacy: 1	> 0.14

Criteria for selection of animals for purchase as replacement	Milk production records: 95, introduction of new pedigree: 46, potential milkability: 34, general animal morphology: 17, animal breed: 1	Milk production records: 20, introduction of new pedigree: 11, potential milkability: 13, general animal morphology: 7, animal breed: 0	> 0.08
Purveyors of replacements to be purchased	Other farms: 155, breeding centres: 5	Other farms: 41, breeding centres: 0	0.25
**Other domestic animals in the farm**			
Adult cattle	Yes: 8, no: 317	Yes: 5, no: 114	0.34
Adult cattle	$\bar{X}$: 9.5 ± 5.0 animals	$\bar{X}$: 36.0 ± 23.0 animals	0.18
Calves	Yes: 28, no: 297	Yes: 11, no: 108	0.84
Calves	$\bar{X}$: 27.0 ± 9.5 animals	$\bar{X}$: 14.0 ± 6.0 animals	0.42
Buffaloes	Yes: 3, no: 322	Yes: 1, no: 118	0.93
Buffaloes	$\bar{X}$: 5.0 ± 3.5 animals	$\bar{X}$: 1 animal	0.44
Pigs	Yes: 36, no: 289	Yes: 14, no: 105	0.84
Pigs	$\bar{X}$: 5.0 ± 1.0 animals	$\bar{X}$: 7.0 ± 2.0 animals	0.18
Rabbits	Yes: 28, no: 297	Yes: 11, no: 108	0.84
Rabbits	$\bar{X}$: 15.0 ± 4.0 animals	$\bar{X}$: 28.5 ± 9.0 animals	0.62
Poultry	Yes: 174, no: 151	Yes: 58, no: 61	0.37
Poultry	$\bar{X}$: 32.5 ± 2.5 animals	$\bar{X}$: 35.0 ± 5.0 animals	0.65
Domestic fowl	Yes: 31, no: 294	Yes: 7, no: 112	0.22
Domestic fowl	$\bar{X}$: 55.0 ± 32.0 animals	$\bar{X}$: 157.0 ± 141.0 animals	0.28
Dogs	Yes: 297, no: 28	Yes: 115, no: 4	0.06
Dogs	$\bar{X}$: 5.0 ± 0.5 animals	$\bar{X}$: 7.0 ± 0.5 animals	0.001
Sheepdogs	Yes: 255, no: 70	Yes: 99, no: 20	0.27
Sheepdogs	$\bar{X}$: 4.0 ± 0.5 animals	$\bar{X}$: 6.0 ± 0.5 animals	0.0001
Cats	Yes: 225, no: 100	Yes: 80, no: 39	0.67
Cats	$\bar{X}$: 5.0 ± 0.5 animals	$\bar{X}$: 5.0 ± 0.5 animals	0.80
Exotic animals	Yes: 0, no: 325	Yes: 0, no: 119	n/a
Horses	Yes: 52, no: 273	Yes: 23, no: 96	0.41
Horses	$\bar{X}$: 2.0 ± 0.5 animals	$\bar{X}$: 2.5 ± 0.5 animals	0.21
Donkeys or mules	Yes: 8, no: 317	Yes: 3, no: 116	0.97
Donkeys or mules	$\bar{X}$: 1.0 ± 0.0 animals	$\bar{X}$: 1.0 ± 0.0 animals	n/a
Other animals	Yes: 0, no: 325	Yes: 0, no: 119	n/a
**Rodents in the farm**			
Visual contacts of the farmer with rodents	Yes: 198, no: 127	Yes: 66, no: 53	0.30
Detection of dead rodents in the farm grounds or within a radius of 500 m of the farm	Yes: 94, no: 231	Yes: 33, no: 86	0.81
Bioindications of rodents in the farm grounds or within a radius of 500 m of the farm	Yes: 304, no: 21	Yes: 110, no: 9	0.68
Description of bioindications of rodents	Rodent faeces: 261, rodent feed: 164	Rodent faeces: 94, rodent feed: 53	> 0.14
Detection of rodents in feed troughs	Yes: 82, no: 243	Yes: 28, no: 91	0.71
Detection of rodents in water pots	Yes: 80, no: 245	Yes: 26, no: 93	0.54
Presence of rodents in fields within a radius of 2 km of the farm	Yes: 196, no: 129	Yes: 76, no: 43	0.50
Rodents identified within a radius of 2 km of the farm	Mice: 193, rats: 2	Mice: 75, rats: 1	> 0.92
Administration of rodenticides	Yes: 127, no: 198	Yes: 50, no: 69	0.58
Annual frequency of administration of rodenticides	$\bar{X}$: 5.5 ± 0.5 occasions	$\bar{X}$: 5.5 ± 1.0 occasions	0.95
**Wildlife mammals**			
Visual contacts of the farmer with wildlife mammals	Yes: 297, no: 28	Yes: 109, no: 10	0.94
Detection of dead wildlife mammals in the farms grounds or within a radius of 500 m of the farm	Yes: 80, no: 245	Yes: 33, no: 86	0.50
Detection of traces of wildlife mammals within a radius of 2 km of the farm	Yes: 184, no: 141	Yes: 68, no: 51	0.92

Detection of faeces of wildlife mammals within a radius of 2 km of the farm	Yes: 185, no: 140	Yes: 67, no: 52	0.91
Detection of damage to cultivations within a radius of 2 km of the farm	Yes: 182, no: 143	Yes: 65, no: 54	0.80
Wildlife mammals identified within a radius of 2 km of the farm	Badger: 31, bear: 19, ferret: 44, fox: 242, hare: 77, jackal: 85, mink: 2, otter: 2, red deer: 8, roe deer: 38, wildboar: 157, wild rabbit: 6, wolf: 129	Badger: 13, bear: 11, ferret: 18, fox: 95, hare: 34, jackal: 36, mink: 3, otter: 1, red deer: 4, roe deer: 19, wildboar: 68, wild rabbit: 5, wolf: 50	> 0.07
Common grazing of sheep / goats with wildlife mammals	Yes: 152, no: 173	Yes: 56, no: 63	0.96
Species of wildlife mammals identified in common grazing with animals of the farm	Red deer: 11, roe deer: 25, wildboar: 75	Red deer: 5, roe deer: 11, wildboar: 26	> 0.29
**Avian wildlife**			
Visual contacts of the farmer with avian wildlife	Yes: 320, no: 5	Yes: 118, no: 1	0.57
Recognition of dead avian wildlife in the farms grounds or within a radius of 500 m of the farm	Yes: 86, no: 239	Yes: 36, no: 83	0.43
Bioindications of avian wildlife in the farms grounds or within a radius of 500 m of the farm	Yes: 212, no: 113	Yes: 78, no: 41	0.95
Recognition of avian wildlife in feed troughs	Yes: 179, no: 146	Yes: 72, no: 47	0.31
Recognition of avian wildlife in water pots	Yes: 182, no: 143	Yes: 74, no: 45	0.24
Presence of nests within the farm buildings	Yes: 131, no: 194	Yes: 52, no: 67	0.52
Presence of eggs in such nests	Yes: 128, no: 197	Yes: 50, no: 69	0.62
Presence of chicks in such nests	Yes: 128, no: 197	Yes: 50, no: 69	0.62
Avian wildlife identified	*Accipiter brevipes*: 10, *Anas platyrhynchos*: 18, *Anser* spp.: 2, *Aquila chrysaetos* ^ *Aquila nipalensis* ^ *Aquila fasciata*: 67, Ardeidae family: 3, *Athene noctua*: 5, *Bubo bubo*: 5, *Carduelis carduelis*: 2, *Ciconia ciconia*: 9, *Columba livia*: 146, *Columba palumbus*: 4, *Corvus corax*: 79, *Corvus cornix*: 10, *Coturnix coturnix*: 8, *Cuculus canorus*: 1, *Cygnus cygnus*: 2, *Erithacus rubecula*: 1, *Falco* spp.: 149, *Fringillia coelebs*: 3, *Galerida crisata*: 2, *Garrulus glandarius*: 26, *Gypaetus barbatus*: 3, *Gyps* spp.: 12, *Hirundo rustica*: 179, Laridae family: 11, *Luscinia megarhynchos*: 1, Passeridae family: 143, *Pelecanus onocrotalus*: 2, *Perdix perdix*: 20, *Phasianus colchicus*: 4, *Phoenicopterus roseus*: 1, *Pica pica*: 266, *Pyrrhocorax graculus*: 8, *Scolopax rusticola*: 17, *Streptopelia decaocto*: 227, *Streptopelia turtur*: 18, *Sturnus vulgaris*: 2, *Turdus merula*: 39, *Turdus philomelos*: 21, *Upupa epops*: 4	*Accipiter brevipes*: 2, *Anas platyrhynchos*: 1, *Anser* spp.: 0, *Aquila chrysaetos* ^ *Aquila nipalensis* ^ *Aquila fasciata*: 30, Ardeidae family: 1, *Athene noctua*: 4, *Bubo bubo*: 2, *Carduelis carduelis*: 1, *Ciconia ciconia*: 1, *Columba livia*: 50, *Columba palumbus*: 3, *Corvus corax*: 46, *Corvus cornix*: 3, *Coturnix coturnix*: 5, *Cuculus canorus*: 0, *Cygnus cygnus*: 0, *Erithacus rubecula*: 1, *Falco* spp.: 55, *Fringillia coelebs*: 0, *Galerida crisata*: 0, *Garrulus glandarius*: 12, *Gypaetus barbatus*: 3, *Gyps* spp.: 5, *Hirundo rustica*: 59, Laridae family: 2, *Luscinia megarhynchos*: 0, Passeridae family: 45, *Pelecanus onocrotalus*: 0, *Perdix perdix*: 15, *Phasianus colchicus*: 3, *Phoenicopterus roseus*: 0, *Pica pica*: 99, *Pyrrhocorax graculus*: 4, *Scolopax rusticola*: 6, *Streptopelia decaocto*: 82, *Streptopelia turtur*: 7, *Sturnus vulgaris*: 1, *Turdus merula*: 16, *Turdus philomelos*: 11, *Upupa epops*: 0	*Corvus cornix*: 0.002, *Perdix perdix*: 0.013, *Hirundo rustica*: 0.022, *Anas platyrhynchos*: 0.027, *Ciconia ciconia*: 0.028, *Gypaetus barbatus*: 0.034, all others: > 0.08

**Hunting**			
Presence of hunters in the area around the farm within a radius of 2 km of the farm	Yes: 284, no: 41	Yes: 113, no: 6	0.022
Weekly frequency of the presence of hunters in the area	3-weekly (2-weekly – 7-weekly) [6]	3-weekly (2-weekly – 7-weekly)	0.35
Description of hunted avian species	*Scolopax rusticola*: 226, *Turdus philomelos*: 222, *Anas platyrhynchos*: 65, *Coturnix coturnix*: 9, *Perdix perdix*: 8, *Turdus merula*: 5, *Phasianus colchicus*: 1, *Columba palumbus*: 1, Passeridae family: 1, *Streptopelia turtur*: 3	*Scolopax rusticola*: 89, *Turdus philomelos*: 88, *Anas platyrhynchos*: 20, *Coturnix coturnix*: 3, *Perdix perdix*: 4, *Turdus merula*: 0, *Phasianus colchicus*: 0, *Columba palumbus*: 1, Passeridae family: 0, *Streptopelia turtur*: 0	> 0.13
Description of hunted mammalian species	Hare: 217, wildboar: 192	Hare: 95, wildboar: 78	Hare: 0.047, wildboar: 0.39
Distance from the farm that hunting activity occurs	$\bar{X}$: 1.8 ± 0.2 km	$\bar{X}$: 1.2 ± 0.2 km	0.043
Months every year during which hunting activity occurs	$\bar{X}$: 4.5 ± 0.1 months	$\bar{X}$: 4.5 ± 0.1 months	0.50
Hunting activity of the farmer	Yes: 52, no: 273	Yes: 14, no: 105	0.27
Length of hunting activity of the farmer	$\bar{X}$: 20 ± 1 years	$\bar{X}$: 18 ± 2 years	0.55
**PRODUCTION CHARACTERISTICS**			
Month of the start of the lambing / kidding season	October (January – December)	October (January – December)	0.11
Usual month of the start of the milking period	October (January – December)	November (January – December)	0.08
Usual month of the end of the milking period	July (January – December)	August (March – December)	0.06
Annual milk quantity per animal obtained during the preceding milking period	$\bar{X}$: 207 ± 5 L	$\bar{X}$: 201 ± 10 L	0.55
Total number of lambs / kids born per female animal during the preceding lambing / kidding season	$\bar{X}$: 1.33 ± 0.1 lambs	$\bar{X}$: 1.30 ± 0.2 kids	0.15
Total number of lambs / kids sold per female animal during the preceding season	$\bar{X}$: 0.72 ± 0.2 lambs	$\bar{X}$: 0.70 ± 0.8 kids	0.68
Average age of lambs / kids at slaughter	$\bar{X}$: 50 ± 1 days	$\bar{X}$: 65 ± 3 days	< 0.0001
Average live bodyweight of lambs / kids at slaughter	$\bar{X}$: 17.5 ± 0.2 kg	$\bar{X}$: 16.6 ± 0.4 days	0.043
Average carcass weight of these at slaughter	$\bar{X}$: 10.0 ± 0.1 kg	$\bar{X}$: 9.4 ± 0.2 days	0.012
Record keeping	Yes: 213, no: 112	Yes: 74, no: 45	0.51
Local manufacturing of dairy products	Yes: 201, no: 124	Yes: 92, no: 27	0.002
Objective of local manufacturing of dairy products	Home consumption: 188, sale: 16	Home consumption: 80, sale: 13	Home consumption: 0.031, sale: 0.05
Types of dairy products in local production	Cheese: 186, yoghurt: 81, tarhana: 8, sour milk: 6, ice-cream: 5, cream: 2, pasta: 2, butter: 1, milk-rice pudding: 1	Cheese: 88, yoghurt: 29, tarhana: 1, sour milk: 2, ice-cream: 5, cream: 1, pasta: 2, butter: 3, milk-rice pudding: 1	Butter: < 0.0001, all others: > 0.06

**HEALTH MANAGEMENT**			
**Health parametres**			
The two health problems in lambs / kids considered to be of the higher importance	Abscesses: 2, acidosis: 1, arthritis - encephalitis: 2, brucellosis: 0, clostridial infection: 24, coliform infections: 12, contagious ecthyma: 25, diarrhoea: 233, endoparasitic infections: 8, injuries: 2, *Listeria* infections: 1, navel infection: 1, paratuberculosis: 1, pica: 1, pneumonia: 89, selenium deficiency: 10, toxicosis: 1	Abscesses: 1, acidosis: 0, arthritis - encephalitis: 1, brucellosis: 1, clostridial infection: 12, coliform infections: 11, contagious ecthyma: 4, diarrhoea: 83, endoparasitic infections: 11, injuries: 0, *Listeria* infections: 0, navel infection: 0, paratuberculosis: 1, pica: 0, pneumonia: 27, selenium deficiency: 1, toxicosis: 1	endoparasitic infections: 0.0009, selenium deficiency: 0.008, contagious ecthyma: 0.034, all others: > 0.15
Total cases of these two health problems in lambs / kids during the preceding season	$\bar{X}$: 56 ± 5.5 cases	$\bar{X}$: 45 ± 7 cases	0.26
Incidence rate of these two health problems in lambs / kids during the preceding season	13.0% (12.3% - 13.6%) [7]	14.6% (13.7% - 15.6%)	0.043
The two health problems in replacement animals considered to be of the higher importance	Abortion: 12, acidosis: 2, brucellosis: 1, caseous lymphadenitis: 3, cerebral coenurosis: 44, clostridial infections: 34, contagious agalactia: 3, contagious ecthyma: 1, diarrhoea: 27, endoparasitic infections: 7, enzootic intranasal tumour: 1, lameness: 4, *Listeria* infections: 1, mastitis: 12, paratuberculosis: 9, pneumonia: 53, pregnancy toxaemia: 1, vaginal prolapse: 2	Abortion: 7, acidosis: 3, brucellosis: 0, caseous lymphadenitis: 3, cerebral coenurosis: 7, clostridial infections: 7, contagious agalactia: 1, contagious ecthyma: 1, diarrhoea: 8, endoparasitic infections: 1, enzootic intranasal tumour: 2, lameness: 2, *Listeria* infections: 0, mastitis: 6, paratuberculosis: 7, pneumonia: 13, pre nancy toxaemia: 0, vaginal prolapse: 0	acidosis: 0.002, cerebral coenurosis: 0.013, caseous lymphadenitis: 0.033, all others > 0.05
Total cases of these two health problems in replacement animals during the preceding season	$\bar{X}$: 11 ± 3 cases	$\bar{X}$: 9 ± 2 cases	0.68
Incidence rate of these two health problems in replacement animals during the preceding season	20.0% (15.4% - 24.1%)	26.5% (22.6% - 28.9%)	0.48
The two health problems in adult animals considered to be of the higher importance	Abortion: 17, acidosis: 2, brucellosis: 0, cerebral coenurosis: 5, clostridial infections: 17, contagious agalactia: 19, contagious ecthyma: 2, copper toxicosis: 2, diarrhoea: 8, endoparasitic infections: 6, enzootic intranasal tumour: 1, external myiosis: 0, hypervitaminosis A: 1, hypocalcaemia: 1, injuries: 0, lameness: 20, Lentivirus infections: 6, *Listeria* infections: 3, mange: 1, mastitis: 215, *Oestrus ovis* infestation: 1, paratuberculosis: 9, pneumonia: 57, pregnancy toxaemia: 3, scrapie: 10, self-suckling: 0, sudden death: 1, toxicosis:	Abortion: 4, acidosis: 0, brucellosis: 1, cerebral coenurosis: 0, clostridial infections: 9, contagious agalactia: 5, contagious ecthyma: 1, copper toxicosis: 0, diarrhoea: 3, endoparasitic infections: 0, enzootic intranasal tumour: 1, external myiosis: 1, hypervitaminosis A: 1, hypocalcaemia: 0, injuries: 1, lameness: 5, Lentivirus infections: 3, *Listeria* infections: 2, mange: 0, mastitis: 51, *Oestrus ovis* infestation: 0, paratuberculosis: 23, pneumonia: 17, pregnancy toxaemia: 0, scrapie: 0, self-suckling: 1, sudden death: 0, toxicosis:	mastitis: < 0.0001, paratuberculosis: < 0.0001, all others: > 0.05

	5, udder oedema: 1, wasting disease: 2	3, udder oedema: 0, wasting disease: 0	
Total cases of these two health problems in adult animals during the preceding season	$\bar{X}$: 26 ± 3.5 cases	$\bar{X}$: 15 ± 2.5 cases	0.07
Incidence rate of these two health problems in adult animals during the preceding season	8.0% (7.2% - 8.7%)	6.3% (5.8% - 6.8%)	0.004
Total deaths, of any cause, in adult animals during the preceding season	$\bar{X}$: 17 ± 1.5 cases	$\bar{X}$: 14 ± 1 cases	0.18
Incidence rate of total deaths, of any cause, in adult animals during the pre-ceding season	5.2% (5.0% - 5.5%)	5.9% (5.8% - 6.0%)	0.023
Collaboration with a veterinarian	Yes: 283, no: 42	Yes: 101, no: 18	0.55
Means of calculating live bodyweight for the administration of pharmaceutical products	Estimation: 252, weighing: 73	Estimation: 93, weighing: 26	0.96
Routine overdosing (compared to dose prescribed) of pharmaceuticals	Yes: 61, no: 264	Yes: 26, no: 93	0.47
Use of laboratory diagnostic examinations	Yes: 133, no: 192	Yes: 52, no: 67	0.60
In samples of milk	Yes: 70, no: 63	Yes: 25, no: 27	0.58
In samples of blood	Yes: 99, no: 34	Yes: 39, no: 13	0.94
In samples of faeces	Yes: 35, no: 98	Yes: 16, no: 36	0.54
Total visits made annually by veterinarians to the farm during the preceding season	$\bar{X}$: 7 ± 0.5 occasions	$\bar{X}$: 7 ± 0.5 occasions	0.86
Reasons for the visits of the veterinarians	Administration of pharmaceutical treatments: 115, feedstuff evaluation: 6, overall appraisal of flock: 163, pregnancy diagnosis: 42, vaccinations: 229	Administration of pharmaceutical treatments: 42, feedstuff evaluation: 2, overall appraisal of herd: 61, pregnancy diagnosis: 8, vaccina ions: 90	vaccinations: 0.030, pregnancy diagnosis: 0.038, all others: > 0.30
Maintenance of prescribed withdrawal periods after administration of pharmaceuticals	Yes: 321, no: 4	Yes: 117, no: 2	0.72
Evaluation of ammonia concentration within the buildings	Yes: 4, no: 321	Yes: 1, no: 118	0.73
Animal deaths from attacks by other animals	Yes: 118, no: 207	Yes: 70, no: 49	< 0.0001
Overall frequency of animal deaths by other animals during the preceding season	Monthly (Monthly – Less frequently than yearly)	Monthly (Monthly – Less frequently than yearly)	0.54
Species of animals that caused animal deaths during the preceding season	Bears: 6, crows: 1, eagles: 1, farm dogs: 23, ferrets: 1, foxes: 8, jackals: 21, wild cats: 1, wolves: 64	Bears: 3, crows: 1, eagles: 1, farm dogs: 3, ferrets: 0, foxes: 3, jackals: 20, wild cats: 2, wolves: 43	farm dogs: 0.008, jackals: 0.042, all others: > 0.15
Animal deaths from natural disasters	Yes: 32, no: 293	Yes: 2, no: 97	0.014
**Diseases of adult animals – mastitis**			
Overall incidence rate during the preceding season	3.9% (3.8% - 4.0%)	2.8% (2.6% - 3.0%)	< 0.0001
Sample collection and testing for diagnostic purposes	Yes: 103, no: 169	Yes: 35, no: 36	0.08
Treatment	Yes: 270, no: 2	Yes: 71, no: 0	0.47
Antibiotics used for treatment	Amoxicillin: 11, ampicillin: 1, cephalosporins: 10, cloxacillin: 4, enrofloxacin: 7, florfenicol: 2, gentamicin: 4, lincomycin: 7, marbofloxacin: 6, oxytetracycline: 60, penicillin: 218, spectinomycin: 4, streptomycin: 200, tylosin: 11	Amoxicillin: 3, ampicillin: 1, cephalosporins: 1, cloxacillin: 3, enrofloxacin: 3, florfenicol: 0, gentamicin: 0, lincomycin: 3, marbofloxacin: 0, oxytetracycline: 20, penicillin: 53, spectinomycin: 2, streptomycin: 50, tylosin: 4	cloxacillin: 0.019, all others: > 0.11

Route for administration of antimicrobials	Systemic: 240, intramammary: 19, systemic & intramammary: 11	Systemic: 63, intramammary: 8, systemic & intramammary: 0	0.12
**Diseases of adult animals – abortion**			
Overall incidence rate during the preceding season	2.0% (1.9% - 2.1%)	2.5% (2.7% - 2.9%)	< 0.0001
Sample collection and testing for diagnostic purposes	Yes: 40, no: 114	Yes: 20, no: 37	0.19
Types of samples collected for testing	Blood: 30, abortive material: 15, milk: 4	Blood: 15, abortive material: 5, milk: 2	> 0.25
Antibiotics used for treatment	Cephalosporins: 1, lincomycin: 1, oxytetracycline: 61, penicillin: 9, streptomycin: 3, tylosin: 1	Cephalosporins: 1, lincomycin: 1, oxytetracycline: 37, penicillin: 2, streptomycin: 0, tylosin: 0	penicillin: 0.011, all others: > 0.12
Collection of aborted material for safe disposal	Yes: 41, no: 113	Yes: 21, no: 36	0.15
**Diseases of adult animals – pregnancy toxaemia**			
Overall incidence rate during the preceding season	0.5% (0.4% - 0.6%)	0.2% (0.1% - 0.3%)	< 0.0001
Treatment performed	Penicillin: 3, streptromycin: 3, ciprofloxacin: 1, dextrose: 13, oxytetracycline: 1, dexamethasone: 1, calcium: 32, propylene glycol: 11, vitamins: 12, molasses: 3, selenium: 1, trace minerals: 2, sodium: 2, nutritional change: 1	Penicillin: 0, streptromycin: 0, ciprofloxacin: 0, dextrose: 0, oxytetracycline: 1, dexamethasone: 0, calcium: 5, propylene glycol: 2 vitamins: 1, molasses: 0, selenium: 0, trace minerals: 0, sodium: 0, nutritional change: 0	dextrose: 0.024, all others: >0.07
**Diseases of adult animals – lameness**			
Overall incidence rate during the preceding season	2.8% (2.7% - 2.9%)	1.2% (1.1% - 1.4%)	< 0.0001
Treatment performed	Lincomycin: 57, oxytetracycline: 24, cephalosporins: 16, tylosin: 4, penicillin: 2, streptomycin: 0, spectinomycin: 2, prednisolone: 0, dexamethasone: 1, quinolones: 1, non-steroidal anti-inflammatory drugs: 1, alamycin: 1, footbathing in copper / zinc solution: 33, foot pairing: 10, diesel bathing: 3, acid-based solutions: 1	Lincomycin: 12, oxytetracycline: 8, cephalosporins: 4, tylosin: 0, penicillin: 1, streptomycin: 1, spectinomycin: 0, prednisolone: 1, dexamethasone: 0, quinolones: 0, non-steroidal anti-inflammatory drugs: 0, alamycin: 0, footbathing in copper / zinc solution: 3, foot pairing: 0, diesel bathing:, acid-based solutions: 0	lincomycin: 0.0001, foot pairing: 0.001, footbathing in copper / zinc solution: 0.030, oxytetracycline: 0.048, all others > 0.12
**Diseases of adult animals – mange**			
Overall incidence rate during the preceding season	1.4% (1.3% - 1.5%)	0.1% (0.1% - 0.1%)	< 0.0001
Treatment performed	Ivermectin: 24, eprinomectin: 8, spectinomycin: 1, moxidectin: 3, various lay 'medications': 3	Ivermectin: 8, eprinomectin: 2, spectinomycin: 0, moxidectin: 0, various lay 'medications': 0	> 0.12
**Diseases of adult animals – obstetrical cases**			
Overall incidence rate during the preceding season	1.0% (0.9% - 1.1%)	0.6% (0.7% - 0.8%)	< 0.0001
Call for veterinary support	Yes: 69, no: 67	Yes: 24, no: 12	0.09
Person who performed manipulations	Veterinarian: 68, farmer: 97, non-veterinary staff: 3	Veterinarian: 22, farmer: 25, non-veterinary staff: 0	> 0.11
**Diseases of young animals – respiratory problems**			
Overall incidence rate during the preceding season	1.4% (1.3% - 1.5%)	1.1% (1.0% - 1.2%)	< 0.0001
Treatment performed	Yes: 71, no: 25	Yes: 17, no: 4	0.99

Antibiotics used for treatment	Amoxicillin: 2, ampicillin: 3, cephalosporins: 3, enrofloxacin: 7, erythromycin: 1, gentamicin: 1, lincomycin: 2, marbofloxacin: 1, oxytetracycline: 40, penicillin: 9, spectinomycin: 2, streptomycin: 3, tilmicosin: 1, tulathromycin: 12, tylosin: 8	Amoxicillin: 0, ampicillin: 2, cephalosporins: 0, enrofloxacin: 2, erythromycin: 0, gentamicin: 0, lincomycin: 0, marbofloxacin: 1, oxytetracycline: 7, penicillin: 3, spectinomycin: 0, streptomycin: 1, tilmicosin: 0, tulathromycin: 5, tylosin: 1	> 0.09
**Diseases of young animals – diarrhoea**			
Overall incidence rate during the preceding season	7.9% (7.8% - 8.0%)	10.4% (10.0% - 10.7%)	< 0.0001
Treatment performed	Yes: 169, no: 25	Yes: 64, no: 5	0.21
Antibiotics used for treatment	Amoxicillin: 35, ampicillin: 4, cephalosporins: 3, cloxacillin: 2, colistin: 1, enrofloxacin: 15, gentamicin: 12, lincomycin: 4, neomycin: 2, oxytetracycline: 46, penicillin: 25, spectinomycin: 13, streptomycin: 17, sulfonamides: 6, tylosin: 13	Amoxicillin: 13, ampicillin: 0, cephalosporins: 0, cloxacillin: 0, colistin: 1, enrofloxacin: 0, gentamicin: 6, lincomycin: 2, neomycin: 0, oxytetracycline: 19, penicillin: 11, spectinomycin: 5, streptomycin: 5, sulfonamides: 4, tylosin: 1	enrofloxacin: 0.014, all others: > 0.16
**Management practices**			
Reproductive management	No hormonal control: 217, administration of melatonin: 23, administration of progestogens: 89	No hormonal control: 99, administration of melatonin: 5, administration of progestogens: 16	0.0007
Duration of mating period	2 (1 – 12) months	2 (1 – 12) months	0.09
Changes of rams / bucks into the ewes / does during the mating period	Yes: 92, no: 233	Yes: 25, no: 94	0.12
Castration of lambs / kids kept for fattening	Yes: 1, no: 324	Yes: 0, no: 119	0.54
Use of vasectomies	Yes: 0, no: 325	Yes: 0, no: 119	n/a
Use of artificial insemination	Yes: 0, no: 325	Yes: 0, no: 119	n/a
Use of embryo transfer	Yes: 0, no: 325	Yes: 0, no: 119	n/a
Use of ultrasound for pregnancy diagnosis	Yes: 119, no: 206	Yes: 20, no: 99	< 0.0001
Nutritional modifications before the lambing period	Yes: 229, no: 96	Yes: 68, no: 51	0.008
Beginning of the mating period for ewes / does	May (February–December)	June (January–December)	< 0.0001
Duration of the mating period for ewes / does	2 (1 – 12) months	2 (1 – 12) months	0.09
Beginning of the mating period for ewe-lambs and doelings	August (January–December)	August (January–December)	0.07
Duration of the mating period for ewe-lambs and doelings	1 (1 – 9) months	1 (1 – 6) months	0.006
Grouping of pregnant females during the final stage of pregnancy	Yes: 214, no: 111	Yes: 69, no: 50	0.13
Induction of lambing	Yes: 5, no: 320	Yes: 1, no: 118	0.57
Newborn care and specific monitoring	Yes: 293, no: 32	Yes: 109, no: 10	0.65
Maintenance of a colostrum bank	Yes: 46, no: 279	Yes: 12, no: 107	0.26
Lamb / kid fostering to female animals other than their dams	Yes: 112, no: 213	Yes: 86, no: 33	< 0.0001

Reasons for doing this practice	Death of dam: 39, improving nutrition of lambs: 1, inadequate milk availability by dam: 14, increased number of lambs: 112, stimulation of milk production in ewe that aborted: 31	Death of dam: 18, improving nutrition of kids: 3, inadequate milk availability by dam: 5, increased number of kids: 54, stimulation of milk production in doe that aborted: 9	increased number of lambs / kids: < 0.0001, stimulation of milk production in female that aborted: 0.001, death of dam: 0.016, all others: > 0.06
Administration of a lamb- / kid-specific diet	Yes: 226, no: 99	Yes: 79, no: 40	0.52
Age for lamb / kid removal from their dams	$\bar{X}$: 50 ± 1 days	$\bar{X}$: 65 ± 3 days	< 0.0001
Daily number of milking sessions	2 (1 – 3)	2 (1 – 3)	0.0001
Method for drying-off at the end of the lactation period	Abrupt: 10, progressive: 315	Abrupt: 5, progressive: 114	0.56
Duration of the dry-period	$\bar{X}$: 3.0 ± 0.1 months	$\bar{X}$: 2.9 ± 0.1 months	0.84
Seasonal transfer of animals to other site	Yes: 49, no: 276	Yes: 28, no: 91	0.037
Means of animal transfer between sites	Walking: 40, truck: 11	Walking: 22, truck: 8	> 0.28
Distance between sites	$\bar{X}$: 32 ± 3 km	$\bar{X}$: 32 ± 5 km	0.99
Nights out during transfer between sites	$\bar{X}$: 1.8 ± 0.1 overnights	$\bar{X}$: 1.8 ± 0.1 overnights	0.86
Post-mortem examination of animals that die	Yes: 171, no: 154	Yes: 64, no: 55	0.83
Disposal of carcasses from dead animals	Burying: 183, disposal by knackers: 3, drop-off at water streams: 35, drop-off away: 52, feeding to birds: 1, feeding to dogs: 49, incineration: 28, slaughterhouse: 0	Burying: 53, disposal by knackers: 3, drop-off at water streams: 19, drop-off away: 23, feeding to birds: 0, feeding to dogs: 29, incineration: 10, slaughterhouse: 1	Feeding to dogs: 0.011, burying: 0.014, all others: > 0.06
Reporting to the farming insurance agency	Yes: 93, no: 232	Yes: 37, no: 82	0.61
Compensation by the farming insurance agency	Yes: 16, no: 77	Yes: 8, no: 29	0.56
Manure management	Spread to fields: 320, disposal: 5, sale: 1	Spread to fields: 109, disposal: 7, sale: 3	< 0.007
Security availability at the farm	Yes: 214, no: 111	Yes: 65, no: 54	0.03
Duration of security attendance	Continuous: 210, regular visits: 4	Continuous: 65, regular visits: 0	0.27
Farm security	Alarm: 53, light wire fence: 68, personnel: 5, stoned wall: 1, strong wire fence: 93	Alarm: 13, light wire fence: 28, personnel: 1, stoned wall: 0, strong wire fence: 26	Light wire fence: 0.047, all others: > 0.19
Availability of disinfectant at entrance ditch	Yes: 1, no: 324	Yes: 0, no: 119	0.54
**Vaccinations**			
Against *Chlamydia* infection	Yes: 130, no: 195	Yes: 39, no: 80	0.16
Description of the most common schedule:	Use in ewe-lambs: 98	Use in doelings: 29	0.90
Against *Toxoplasma* infection	Yes: 0, no: 325	Yes: 0, no: 119	n/a
Description of the most common schedule:	n/a	n/a	n/a
Against *Brucella* infection	Yes: 301, no: 0	Yes: 106, no: 0	n/a
Description of the most common schedule:	Use in females kept for replacement: 301	Use in females kept for replacement: 106	n/a
Against clostridial infection	Yes: 316, no: 9	Yes: 118, no: 1	0.23
Description of the most common schedule:	Use in pregnant females one month before expected start of lambing season: 275	Use in pregnant females one month before expected start of kidding season: 105	0.58

Against mastitis	Yes: 126, no: 199	Yes: 34, no: 85	0.047
Description of the most common schedule:	Use in pregnant females during gestation: 103	Use in pregnant females during gestation: 31	0.12
Against contagious agalactia	Yes: 186, no: 139	Yes: 65, no: 54	0.62
Description of the most common schedule:	Use in pregnant females three months before expected start of lambing season: 38	Use in pregnant females three months before expected start of kidding season: 18	0.23
Against bacterial respiratory infections	Yes: 144, no: 181	Yes: 39, no: 80	0.028
Description of the most common schedule:	Use in association with vaccine against clostridial infections: 129	Use in association with vaccine against clostridial infections: 32	0.20
Against orf	Yes: 3, no: 322	Yes: 1, no: 118	0.93
Description of the most common schedule:	Use under special administration licence: 3	Use under special administration licence: 1	n/a
Against paratuberculosis	Yes: 11, no: 314	Use: 31, no: 88	< 0.0001
Description of the most common schedule:	Use in ewe-lambs: 11	Use in doelings: 31	n/a
Against foot-rot	Yes: 5, no: 320	Yes: 0, no: 119	0.17
Description of the most common schedule:	Use in cases of disease in the farm: 5	n/a	n/a
**Antiparasitic administrations – Anthelmintic treatments to small ruminants**			
Administration of anthelmintics to sheep / goats in the farm	Yes: 322, no: 3	Yes: 117, no: 2	0.50
Timing of administration within the annual production cycle	Before the mating season: 24, continuously: 2, final stage of pregnancy: 221, initial stage of dry period: 170, 1st-2nd month of lactation period: 89, 3rd-6th month of lactation period: 32	Before the mating season: 7, continuously: 0, final stage of pregnancy: 79, initial stage of dry period: 74, 1st-2nd month of lactation period: 33, 3rd-6th month of lactation period: 10	Initial stage of dry period: 0.026, all others: > 0.29
Anthelmintics administered	Albendazole: 201, closantel: 11, eprinomectin: 85, fenbendazole: 46, ivermectin: 180, levamisole: 8, moxidectin: 15, netobimin: 30, oxfendazole: 19, rafoxanide: 15	Albendazole: 75, closantel: 5, eprinomectin: 41, fenbendazole: 23, ivermectin: 65, levamisole: 2, moxidectin: 3, netobimin: 8, oxfendazole: 6, rafoxanide: 4	> 0.10
Pharmaceutical form administered	Tablet: 221, injectable solution: 183, oral drench: 150, pour-on: 10, premix: 2	Tablet: 88, injectable solution: 71, oral drench: 47, pour-on: 9, premix: 0	Pour-on: 0.037, all others: > 0.18
Use of environmental applications for helminth control	Yes: 157, no: 168	Yes: 50, no: 69	0.24
Which ones	Disinfection: 157	Disinfection: 50	n/a
**Antiparasitic administrations – Ectoparasiticide treatments to small ruminants**			
Administration of ectoparasiticides to sheep / goats in the farm	Yes: 109, no: 216	Yes: 69, no: 50	< 0.0001
Timing of administration within the annual production cycle	Before the mating season: 7, final stage of pregnancy: 60, initial stage of dry-period: 49, 1st-2nd month of lactation period: 8, 3rd-6th month of lactation period: 2	Before the mating season: 5, final stage of pregnancy: 38, initial stage of dry-period: 31, 1st-2nd month of lactation period: 8, 3rd-6th month of lactation period: 5	3rd-6th month of lactation period: 0.002, all others: > 0.15
Ectoparasiticides administered	Cypermethrin: 47, deltamethrin: 51, eprinomectin: 2, ivermectin: 1, phoxim: 26	Cypermethrin: 31, deltamethrin: 38, eprinomectin: 1, ivermectin: 1, phoxim: 16	> 0.30

Pharmaceutical form administered	Pour-on solution: 93, solution for spraying: 25, injectable solution: 1	Pour-on solution: 58, solution for spraying: 16, injectable solution: 1	0.46
Use of environmental applications for ectoparasite control	Yes: 10, no: 315	Yes: 5, no: 114	0.56
Which ones	Spraying: 10	Spraying: 5	n/a
**Antiparasitic administrations – Antiparasitic treatments to dogs in the farm**			
Administration of antiparasitics to dogs in the farm	Yes: 256, no: 41	Yes: 95, no: 20	0.36
Antiparasitics used	Febantel: 236, fluralaner: 2, ivermectin: 20, isoxazoline: 14, milbemycin: 15, praziquantel: 237, pyrantel: 236	Febantel: 85, fluralaner: 3, ivermectin: 8, isoxazoline: 5, milbemycin: 8, praziquantel: 87, pyrantel: 85	> 0.09
Pharmaceutical form administered	Injectable solution: 6, tablet: 253	Injectable solution: 3, tablet: 95	> 0.15
**Other health management practices**			
Application of disinfections in the farm	Yes: 296, no: 29	Yes: 102, no: 17	0.10
Annual frequency of systemic disinfections in the farm	$\bar{X}$: 3.6 ± 0.2 occasions	$\bar{X}$: 4.3 ± 0.5 occasions	0.08
Administration of oxytetracycline to the pregnant animals	Yes: 156, no: 169	Yes: 60, no: 59	0.65
Administration of selenium to pregnant animals	Yes: 97, no: 228	Yes: 37, no: 82	0.80
Administration of selenium to newborn animals	Yes: 228, no: 97	Yes: 67, no: 52	0.006
Administration of 'dry-ewe' treatment at the end of the lactation period	Yes: 53, no: 272	Yes: 15, no: 104	0.34
Use of teat disinfection after milking	Yes: 52, no: 273	Yes: 12, no: 107	0.12
Use of teat spraying after milking	Yes: 0, no: 325	Yes: 0, no: 119	n/a
Weekly frequency of changing teat disinfectant in the cup	$\bar{X}$: 1.6 ± 0.1 occasions	$\bar{X}$: 1.8 ± 0.1 occasions	0.45
Foot care	Yes: 224, no: 101	Yes: 72, no: 47	0.10
Annual frequency of foot care	$\bar{X}$: 1.4 ± 0.1 occasions	$\bar{X}$: 1.3 ± 0.1 occasions	0.37
Shearing	Yes: 319, no: 6	Yes: 102, no: 17	< 0.0001
Shearing equipment used	Shearing shears: 182, shearing machine: 137	Shearing shears: 53, shearing machine: 49	0.37
Recording of births – maintenance of a lambing / kidding book	Yes: 182, no: 143	Yes: 53, no: 66	0.032
Disinfection of navel stumps in newborns	Yes: 213, no: 112	Yes: 65, no: 54	0.035
Tail docking in newborns	Yes: 244, no: 81	Yes: 34, no: 85	< 0.0001
Routine administration of antimicrobials in newborns	Yes: 65, no: 260	Yes: 29, no: 90	0.32
Antimicrobials administered	Amoxicillin: 17, ampicillin: 1, cephalosporins: 0, cloxacillin: 1, enrofloxacin: 5, gamithromycin: 1, gentamicin: 3, lincomycin: 1, oxytetracycline: 36, penicillin: 12, spectinomycin: 2, streptomycin: 5, sulfonamides: 0, tulathromycin: 2, tylosin: 1	Amoxicillin: 10, ampicillin: 2, cephalosporins: 2, cloxacillin: 0, enrofloxacin: 3, gamithromycin: 0, gentamicin: 1, lincomycin: 0, oxytetracycline: 15, penicillin: 6, spectinomycin: 0, streptomycin: 4, sulfonamides: 1, tulathromycin: 0, tylosin: 0	> 0.19
Maintenance of quarantine period for new animals into the farm	Yes: 211, no: 114	Yes: 61, no: 58	0.009
Isolation of sick animals	Yes: 276, no: 49	Yes: 98, no: 21	0.51
**Vectors**			
Presence of spots suitable for reproduction of vectors	Yes: 223, no: 102	Yes: 83, no: 36	0.82
Types of spots identified	Muddy spots inside or outside the buildings, near the water troughs: 194, spots of wet manure: 30, ditches with manure: 0	Muddy spots inside or outside the buildings, near the water troughs: 74, spots of wet manure: 10, ditches with manure: 0	> 0.30

Distance of spots from farm	> 50 m: 128, 50-500 m: 58, > 500 m: 37	> 50 m: 57, 50-500 m: 17, > 500 m: 9	> 50 m: 0.036, all others: > 0.10
**NUTRITION**			
Grazing practiced	Yes: 281, no: 44	Yes: 112, no: 7	0.025
Duration of grazing during the winter	$\bar{X}$: 3.6 ± 0.1 months	$\bar{X}$: 4.4 ± 0.2 months	0.002
Distance from farm of area grazed during the winter	$\bar{X}$: 1.3 ± 0.1 km	$\bar{X}$: 2.2 ± 0.2 km	0.0009
Duration of grazing during the summer	$\bar{X}$: 4.9 ± 0.1 months	$\bar{X}$: 5.0 ± 0.2 months	0.82
Distance from farm of area grazed during the summer	$\bar{X}$: 1.6 ± 0.1 km	$\bar{X}$: 2.4 ± 0.2 km	0.004
Type of graze area	Forest: 18, hay: 4, meadow: 204, olive trees: 2, scrub pasture: 83, wetland: 16	Forest: 23, hay: 5, meadow: 59, olive trees: 0, scrub pasture: 58, wetland: 5	Meadow, scrub pasture, forest: < 0.0001, all others: > 0.26
Common grazing for sheep and goats	Yes: 48, no: 277	Yes: 40, no: 79	< 0.0001
Provision of hay as fodder to animals	Yes: 324, no: 1	Yes: 116, no: 3	0.029
Total quantity of hay consumed during the preceding season	$\bar{X}$: 84 ± 5 tonnes	$\bar{X}$: 46 ± 7 tonnes	0.0003
Plants included in hay consumed by animals	*Avena sativa*: 25, *Euphorbia pulcherrima*: 1, *Hordeum vulgare*: 22, *Lolium*: 15, *Medicago sativa*: 10, *Pisum sativum*: 7, *Polygonum aviculare*: 2, *Trifolium*: 295, *Triticum*: 5, *Vicia*: 25	*Avena sativa*: 21, *Euphorbia pulcherrima*: 0, *Hordeum vulgare*: 8, *Lolium*: 1, *Medi cago sativa*: 2, *Pisum sativum*: 2, *Polygonum aviculare*: 0, *Trifolium*: 106, *Triticum*: 0, *Vicia*: 7	*Lolium*, *Medicago sativa*, *Pisum sativum*, *Triticum*: < 0.0001, all others: > 0.09
Hay type	Roughage: 323, pelleted: 8	Roughage: 115, pelleted: 1	> 0.25
Origin of hay	Own production: 193, purchase: 206	Own production: 55, purchase: 81	Own production: 0.005, purchase: 0.11
Provision of straw to animals	Yes: 258, no: 67	Yes: 78, no: 41	0.003
Provision of silage to adult animals	Yes: 72, no: 253	Yes: 18, no: 101	0.10
Provision of silage to young animals	Yes: 18, no: 307	Yes: 4, no: 115	0.35
Total quantity of silage consumed during the preceding season	$\bar{X}$: 142 ± 31 tonnes	$\bar{X}$: 98 ± 35 tonnes	0.50
Origin of silage	Own production: 36, purchase: 42	Own production: 10, purchase: 12	> 0.25
Provision of finished feed to animals	Yes: 304, no: 21	Yes: 103, no: 16	0.018
Origin of concentrate	Own production: 36, purchase: 42	Own production: 10, purchase: 12	> 0.25
Provision of finished feed (concentrate) to animals throughout the year	Yes: 304, no: 21	Yes: 103, no: 16	0.018
Finished feed (concentrate) form provided to adult animals	Flakes: 4, mash: 113, pellets: 103, small pellets: 92	Flakes: 0, mash: 33, pellets: 36, small pellets: 38	> 0.10
Provision of finished feed (concentrate) to young animals	Yes: 255, no: 70	Yes: 79, no: 40	0.009
Finished feed (concentrate) type provided to young animals	Flakes: 0, mash: 102, pellets: 82, small pellets: 80	Flakes: 0, mash: 26, pellets: 22, small pellets: 35	Small pellets: 0.017, all others: > 0.12
Total quantity of finished feed (concentrate) consumed during the preceding season	$\bar{X}$: 86 ± 6 tonnes	$\bar{X}$: 66 ± 8 tonnes	0.12
Total quantity of raw materials purchased during the preceding season	$\bar{X}$: 63 ± 5 tonnes	$\bar{X}$: 53 ± 6 tonnes	0.12

Raw materials used by the farm in the diets	Barley: 227, bran: 185,cottonseed meal: 84, maize: 294, soyabean meal: 201: sunflower meal: 78, wheat: 104	Barley: 86, bran: 66, cottonseed meal: 42, maize: 110, soyabean meal: 68: sunflower meal: 28, wheat: 42	cottonseed meal: 0.025, all others: > 0.18
Raw materials purchased by the farm for use in the diets	Barley: 205, bran: 196, cottonseed meal: 100, maize: 258, soyabean meal: 215, sunflower meal: 87, wheat: 87	Barley: 84, bran: 68, cottonseed meal: 49, maize: 105, soyabean meal: 68, sunflower meal: 29, wheat: 41	maize: 0.016, cottonseed meal: 0.020, soyabean meal: 0.040, all others: > 0.05
Premix purchase for use in the diets	Yes: 286, no: 39	Yes: 97, no: 22	0.08
Feed change in animals	Abrupt: 3, progressive: 314	Abrupt: 2, progressive: 114	< 0.0001
Application of hydroponics cultivations	Yes: 4, no: 321	Yes: 1, no: 118	< 0.0001
Water source	Local water board: 211, local water drilling: 103, other: 25	Local water board: 74, local water drilling: 34, other: 16	> 0.26
Use of laboratory examinations for quality testing of feeds and raw material	Yes: 79, no: 246	Yes: 19, no: 100	< 0.0001
Laboratory examinations used	Chemical analysis: 37, microbiological examination: 4, mycotoxin detection: 39, residues analysis: 3	Chemical analysis: 7, microbiological examination: 1, mycotoxin detection: 8, residues analysis: 2	Residues analysis: 0.009, all others: > 0.21
Use of laboratory examinations for quality testing of water	Yes: 52, no: 273	Yes: 13, no: 106	< 0.0001
Laboratory examinations used	Overall assessment: 31, chemical analysis: 14, microbiological examination: 8, mycotoxin detection: 1	Overall assessment: 8, chemical analysis: 6, microbiological examination: 4, mycotoxin detection: 0	> 0.07
Person responsible for nutritional management	Farmer themselves: 198, veterinarian: 96, animal scientist: 81, other farmer: 0	Farmer themselves: 94, veterinarian: 28, animal scientist: 22, other farmer: 0	Farmer themselves: 0.0002, all others: > 0.07
**HUMAN RESOURCES**			
**Farmer**			
Sex	Female: 19, male: 316	Female: 11, male: 108	0.21
Age	$\bar{X}$: 47 ± 1 years	$\bar{X}$: 47 ± 1 years	0.64
Length of previous animal farming experience	$\bar{X}$: 24 ± 1 years	$\bar{X}$: 25 ± 1 years	0.80
Highest level of general education	Primary: 57, secondary: 225, tertiary: 43	Primary: 20, secondary: 89, tertiary: 10	0.35
Professional education	Yes: 54, no: 171	Yes: 12, no: 107	0.002
Professional education	Seminars re farming practice: 34, technological education: 6, meat processing training: 4, dairy processing training: 3, animal science degree: 2, farming analysis: 1	Seminars re farming practice: 9, technological education: 2, meat processing training: 0, dairy processing training: 0,animal science degree: 1, farming analysis: 0	> 0.18

Primary language spoken	Greek: 308, Turkish: 13, Albanian: 2, Bulgarian: 1, Indian: 1	Greek: 110, Turkish: 8, Albanian: 1, Bulgarian: 0, Indian: 0	0.70
Farmer by profession	Yes: 292, no: 33	Yes: 105, no: 14	0.63
Daily period of presence in the farm	$\bar{X}$: 11.5 ± 0.2 hours	$\bar{X}$: 12.3 ± 0.3 hours	0.047
Marital status	Single: 59, married: 166	Single: 28, married: 91	0.58
**Public health**			
Personal opinion regarding occurrence of transmission of diseases from animals to the farmer or members of the family	Yes: 44, no: 281	Yes: 27, no: 92	0.020
Diseases, according to above, for which transmission occurred from animals	Anthrax: 1, brucellosis: 35, contagious ecthyma: 2, flea infestation: 2, fungal (*Trichophyton*) infection: 1, hydatid disease: 1, ophthalmic myiasis: 1, tick infestation: 1	Anthrax: 1, brucellosis: 22, contagious ecthyma: 1, flea infestation: 1, fungal (*Trichophyton*) infection: 1, hydatid disease: 1, ophthalmic myiasis: 1, tick infestation: 0	> 0.28
**Family**			
Total members of the family	$\bar{X}$: 4.3 ± 0.1 persons	$\bar{X}$: 4.2 ± 0.1 persons	0.57
Family tradition in farming	Yes: 283, no: 42	Yes: 104, no: 15	0.93
Total members of the family working at the farm	$\bar{X}$: 2.2 ± 0.0 persons	$\bar{X}$: 2.2 ± 0.0 persons	0.34
**Staff**			
Farm worker employment	Yes: 123, no: 202	Yes: 34, no: 85	0.07
Origin	Greek local: 13, Greek from other part of the country: 6, non-Greek: 111	Greek local: 3, Greek from other part of the country: 2, non-Greek: 32	> 0.20
Age	$\bar{X}$: 34 ± 1 years	$\bar{X}$: 35 ± 1 years	0.37
Previous farming experience at start of employment	Yes: 104, no: 19	Yes: 31, no: 3	0.32
Length of previous animal farming experience	$\bar{X}$: 13 ± 1 years	$\bar{X}$: 13 ± 1 years	0.80

[1] *p*-value for comparison between sheep flocks and goat herds; [2] Management system classified according to the system of the European Food Safety Authority [Scientific opinion on the welfare risks related to the farming of sheep for wool, meat and milk production. *EFSA J. 12*, 3933–4060]; [3] Shaded cells indicate significant ($p < 0.05$) differences between sheep flocks and goat herds for respective parametres; [4] Figures present mean ± standard error of the mean ($\bar{X} \pm \sigma_M$.); [5] Figures present frequency (*n*) for each category within the variable; [6] Figures present median (minimum – maximum) value; [7] Figures present average (95% confidence interval) value.

Table S3. Cumulative findings of all results obtained during field and laboratory examinations in samples collected from 444 small ruminant farms in a countrywide investigation in Greece, classified according to animal species in the farms.

	SHEEP FLOCKS n =325	GOAT HERDS n = 199	p-value [1]
**CLINICAL EXAMINATIONS OF ANIMALS AT THE FARMS**			
Body condition scoring	$\bar{X}$: 2.38 ± 0.02 (scale: 0-5) [3]	$\bar{X}$: 2.54 ± 0.03 (scale: 0-5) [3]	< 0.0001 [2]
**LABORATORY EXAMINATIONS IN BULK-TANK MILK**			
**Somatic cell counting**			
Somatic cell counts	$0.488 \times 10^6 (0.451 \times 10^6–0.529 \times 10^6)$ cells mL^{-1} [4]	0.838×10^6 $(0.759 \times 10^6–0.933 \times 10^6)$ cells mL^{-1}	< 0.0001 [2]
**Microbiological examinations**			
Total bacterial counts	398×10^3 $(331 \times 10^3–479 \times 10^3)$ cfu mL^{-1}	581×10^3 $(447 \times 10^3-741 \times 10^3)$ cfu mL^{-1}.	< 0.0001
Staphylococcal isolation	Yes: 206, no: 119 [5]	Yes: 75, no: 44	0.94
Staphylococcal species identified	*S. auricularis*: 3, *S. capitis*: 6, *S. carnosus*: 2, *S. chromogenes*: 13, *S. cohnii* subsp. *cohnii*: 4, *S. cohnii* subsp. *urealyticum*: 3, *S. epidermidis*: 4, *S. equorum*: 23, *S. haemolyticus*: 22, *S. hominis*: 2, *S. intermedius*: 6, *S. kloosii*: 7, *S. lentus*: 12, *S. lugdunensis*: 11, *S. pasteuri*: 2, *S. pettenkoferi*: 0, *S. saprophyticus*: 4, *S. sciuri*: 3, *S. simulans*: 35, *S. vitulinus*: 3, *S. warneri*: 9, *S. xylosus*: 4	*S. auricularis*: 1, *S. capitis*: 6, *S. carnosus*: 0, *S. chromogenes*: 1, *S. cohnii* subsp. *cohnii*: 1, *S. cohnii* subsp. *urealyticum*: 2, *S. epidermidis*: 1, *S. equorum*: 11, *S. haemolyticus*: 4, *S. hominis*: 0, *S. intermedius*: 1, *S. kloosii*: 3, *S. lentus*: 5, *S. lugdunensis*: 2, *S. pasteuri*: 0, *S. pettenkoferi*: 3, *S. saprophyticus*: 0, *S. sciuri*: 0, *S. simulans*: 9, *S. vitulinus*: 4, *S. warneri*: 2, *S. xylosus*: 2	
Biofilm formation in staphylococcal isolates	Yes: 166, no: 66	Yes: 58, no: 22	0.87
Resistance of staphylococci to ampicillin, azithromycin, cefoxitin, ciprofloxacin, clarithromycin, clindamycin, erythromycin, fosfomycin, fucidic acid, gentamicin, moxifloxacin, mupirocin, oxaxillin, penicillin, rifampicin, teicoplanin, tetracycline, tobramycin, trimethoprim-sulfamethoxazole	Yes: 79, 0, 0, 2, 0, 41, 21, 31, 14, 2, 1, 1, 27, 79, 1, 0, 28, 2, 2, respectively, no: 153, 232, 232, 230, 232, 191, 211, 201, 218, 230, 231, 231, 205, 153, 231, 232, 204, 230, 230, respectively	Yes: 33, 0, 0, 0, 0, 19, 16, 22, 3, 0, 0, 0, 6, 33, 0, 1, 12, 1, 0, respectively, no: 47, 80, 80, 80, 80, 61, 64, 58, 77, 80, 80, 80, 74, 47, 80, 79, 68, 79, 80, respectively	Fosfomycin: 0.002, erythromycin: 0.040, all others: > 0.11
Listeria isolation	Yes: 4, no: 321	Yes: 0, no: 119	0.22
Listeria species identified	*L. ivanovii*: 3, *L. monocytogenes*: 1	n/a	n/a
Resistance of listeria to benzylpenicillin, ampicillin, meropenem, erythromycin, trimethoprim/sulfamethoxazole	Yes: 0, 0, 0, 0, 0, respectively, No: 4, 4, 4, 4, 4, respectively	n/a	n/a
**Composition analysis**			
Fat content	$\bar{X}$: 6.16% ± 0.05%	$\bar{X}$: 4.77% ± 0.44%	0.0005
Protein content	$\bar{X}$: 4.43% ± 0.01%	$\bar{X}$: 3.23% ± 0.30%	0.0008
Lactose content	$\bar{X}$: 4.21% ± 0.02%	$\bar{X}$: 4.74% ± 0.03%	< 0.0001
Added water	$\bar{X}$: 0.68% ± 0.01%	$\bar{X}$: 0.50% ± 0.02%	0.37
**PARASITOLOGICAL EXAMINATIONS IN FAECES**			
Detection of parasitic elements of trematodes	*Dicrocoelium dendriticum*: 54, *Fasciola hepatica*: 2, *Paramphistomum cervi*: 7	*Dicrocoelium dendriticum*: 18, *Fasciola hepatica*: 0, *Paramphistomum cervi*: 1	> 0.19
Detection of parasitic elements of cestodes	*Moniezia* spp.: 61	*Moniezia* spp.: 30	0.08
Detection of parasitic elements of intestinal nematodes	Teladorsagia spp.: 278, *Haemonchus contortus*: 277,	Teladorsagia spp.: 106, *Haemonchus contortus*: 106,	> 0.24

	Ttichostrongylus spp.: 257, *Chabertia* spp.: 206, *Cooperia* spp. 140, *Bunostomum* spp.: 65, *Nematodirus* spp.: 61, *Strongyloides papillosus*: 23, *Trichuris* spp.: 65	*Ttichostrongylus* spp.: 98, *Chabertia* spp.: 81, *Cooperia* spp. 54, *Bunostomum* spp.: 33, *Nematodirus* spp.: 20, *Strongyloides papillosus*: 6, *Trichuris* spp.: 22	
epg counts	$\bar{X}$: 214 ± 13	$\bar{X}$: 219 ± 22	0.77
Detection of parasitic elements of respiratory nematodes	Metastrongylidae family: 58	Metastrongylidae family: 28	0.21
Detection of parasitic elements of protozoa	*Eimeria* spp.: 233, *Giardia* spp.: 106: *Crytptosporidium* spp.: 23	*Eimeria* spp.: 72, *Giardia* spp.: 40: *Crytptosporidium* spp.: 13	*Eimeria* spp.: 0.024, all others > 0.18

[1] *p*-value for comparison between sheep flocks and goat herds; [2] Shaded cells indicate significant ($p < 0.05$) differences between sheep flocks and goat herds for respective parametres; [3] Figures present mean ± standard error of the mean ($\bar{X} \pm \sigma_{M}$.); [4] Figures present mean (95% confidence interval); [5] Figures present frequency (*n*) for each category within the variable.

Table S4. Season of the year during which visits were made to 444 small ruminant farms in a countrywide investigation in Greece.

Season of the year	Number of farms visited (proportion)
Autumn	41 (9.2%)
Winter	110 (24.8%)
Spring	151 (34.0%)
Summer	142 (32.0%)

Table S5. Management system applied in 444 small ruminant farms in a countrywide investigation in Greece in accord with the location of the farms.

Management system applied in farms	Geographical area of the country			
	Central	Islands	North	South
**Sheep flocks (n = 325)**				
Intensive	5.9% [1]	0.9%	5.2%	1.5%
semi-Intensive	22.5%	2.5%	10.8%	7.4%
semi-Extensive	9.8%	4.6%	10.2%	11.1%
Extensive	0.9%	5.2%	0.6%	0.9%
**Goat herds (n = 119)**				
Management system applied in farms	Central	Islands	North	South
Intensive	5.9% [1]	0.9%	0.9%	0.0%
semi-Intensive	5.9%	0.8%	12.6%	5.0%
semi-Extensive	13.5%	6.7%	14.3%	16.8%
Extensive	5.0%	5.0%	2.5%	4.2%

[1]: proportions refer to total farms with the same animal species.

Table S6. Breed of ewes and does in 444 small ruminant farms in a countrywide investigation in Greece in accord with the management applied therein.

Animal breed	Management system applied in farms			
	Intensive	Semi-Intensive	Semi-Extensive	Extensive
**Sheep flocks (n = 325)**				
Assaf (n = 30) [1]	16.6% [1]	56.7%	26.7%	0.0%
Awassi (n = 1)	0.0%	100.0%	0.0%	0.0%
Boutsko (n = 2)	0.0%	0.0%	100.0%	0.0%
Chios (n = 44)	22.7%	45.5%	31.8%	0.0%
Crossbreds (n = 43)	7.0%	39.5%	46.5%	7.0%
Friesarta (n = 12)	25.0%	33.3%	33.3%	8.4%
Friesian (n = 13)	0.0%	46.2%	53.8%	0.0%
Karagouniko (n = 5)	0.0%	60.0%	20.0%	20.0%
Kefallinia (n = 1)	0.0%	0.0%	100.0%	0.0%
Lacaune (n = 95)	21.1%	54.7%	23.2%	1.0%
'Local' [2] (n = 55)	3.7%	21.8%	52.7%	21.8%
Mytilini (n = 18)	5.6%	44.4%	33.3%	16.7%
Sfakia (n = 6)	0.0%	0.0%	33.3%	66.7%
**Goat herds (n = 119)**				
Animal breed	Intensive	Semi-Intensive	Semi-Extensive	Extensive
Alpine (n = 9) [1]	11.1% [1]	11.1%	77.8%	0.0%
Crossbreds (n = 18)	5.5%	16.7%	77.8%	0.0%
Damascus (n = 18)	0.0%	50.0%	44.4%	5.6%
Kefallinia (n = 1)	0.0%	0.0%	100.0%	0.0%
Indigenous Greek (*Capra prisca*) (n = 50)	2.0%	10.0%	54.0%	34.0%
Murciano-Granadina (n = 13)	38.5%	53.8%	7.7%	0.0%
Saanen (n = 5)	20.0%	60.0%	20.0%	0.0%
Skopelos (n = 5)	0.0%	20.0%	40.0%	40.0%

[1]: proportions refer to farms with the same breed of animals; [2]: the term used by the respective farmers to refer to a variety of small-scale breeds, not always related between them, and each one prevailing only in some areas of the country with limited geographic dissemination

Table S7. Breed of ewes and does in 444 small ruminant farms in a countrywide investigation in Greece in accord with the milking mode applied in the farms.

Sheep flocks (n = 325)		
Animal breed	Milking mod applied in the farms	
	Machine-milking	Hand-milking
Assaf (n = 30) [1]	93.3% [1]	6.7%
Awassi (n = 1)	100.0%	0.0%
Boutsko (n = 2)	0.0%	100.0%
Chios (n = 44)	77.3%	22.7%
Crossbreds (n = 43)	72.1%	27.9%
Friesarta (n = 12)	91.7%	8.3%
Friesian (n = 13)	46.2%	53.8%
Karagouniko (n = 5)	80.0%	20.0%
Kefallinia (n = 1)	100.0%	0.0%
Lacaune (n = 95)	96.8%	3.2%
'Local' [2] (n = 55)	56.4%	43.6%
Mytilini (n = 18)	83.3%	16.7%
Sfakia (n = 6)	16.7%	83.3%

Goat herds (n = 119)		
Animal breed	Management system applied in farms	
	Intensive or Semi-intensive	Semi-extensive or Extensive
Alpine (n = 9) [1]	66.7% [1]	33.3%
Crossbreds (n = 18)	83.3%	16.7%
Damascus (n = 18)	55.6%	44.4%
Kefallinia (n = 1)	100.0%	0.0%
Indigenous Greek (*Capra prisca*) (n = 50)	36.0%	64.0%
Murciano-Granadina (n = 13)	84.6%	15.4%
Saanen (n = 5)	80.0%	20.0%
Skopelos (n = 5)	20.0%	80.0%

[1]: proportions refer to farms with the same breed of animals; [2]: the term used by the respective farmers to refer to a variety of small-scale breeds, not always related between them, and each one prevailing only in some areas of the country with limited geographic dissemination

Table S8. Breed of ewes and does in 444 small ruminant farms in a countrywide investigation in Greece in accord with the location of the farms.

Sheep flocks (*n* = 325)				
Animal breed	Geographical area of the country			
	Central	Islands	North	South
Assaf (*n* = 30) [1]	26.7% [1]	3.3%	36.7%	33.3%
Awassi (*n* = 1)	100.0%	0.0%	0.0%	0.0%
Boutsko (*n* = 2)	50.0%	0.0%	0.0%	50.0%
Chios (*n* = 44)	38.7%	6.8%	47.7%	6.8%
Crossbreds (*n* = 43)	58.1%	4.7%	18.6%	18.6%
Friesarta (*n* = 12)	75.0%	0.0%	16.7%	8.3%
Friesian (*n* = 13)	15.4%	0.0%	23.1%	61.5%
Karagouniko (*n* = 5)	80.0%	0.0%	20.0%	0.0%
Kefallinia (*n* = 1)	0.0%	100.0%	0.0%	0.0%
Lacaune (*n* = 95)	47.4%	2.1%	33.7%	16.8%
'Local' [2] (*n* = 55)	27.3%	20.0%	16.3%	36.4%
Mytilini (*n* = 18)	0.0%	94.4%	0.0%	5.6%
Sfakia (*n* = 6)	0.0%	100.0%	0.0%	0.0%
**Goat herds (*n* = 119)**				
Animal breed	Geographical area of the country			
	Central	Islands	North	South
Alpine (*n* = 9) [1]	22.2% [1]	33.4%	22.2%	22.2%
Crossbreds (*n* = 18)	16.7%	0.0%	50.0%	33.3%
Damascus (*n* = 18)	22.2%	5.6%	44.4%	27.8%
Kefallinia (*n* = 1)	0.0%	0.0%	100.0%	0.0%
Indigenous Greek (*Capra prisca*) (*n* = 50)	28.0%	22.0%	22.0%	28.0%
Murciano-Granadina (*n* = 13)	69.2%	0.0%	23.1%	7.7%
Saanen (*n* = 5)	60.0%	0.0%	20.0%	20.0%
Skopelos (*n* = 5)	20.0%	20.0%	40.0%	20.0%

[1]: proportions refer to farms with the same breed of animals; [2]: the term used by the respective farmers to refer to a variety of small-scale breeds, not always related between them, and each one prevailing only in some areas of the country with limited geographic dissemination
